# Serotype epidemiology and case-fatality risk of invasive pneumococcal disease: a nationwide population study from Switzerland, 2012–2022

**DOI:** 10.1080/22221751.2025.2488189

**Published:** 2025-04-01

**Authors:** Werner C. Albrich, Nicolaj Just, Christian Kahlert, Carlo Casanova, Florent Baty, Markus Hilty

**Affiliations:** a Division of Infectious Diseases, Infection Prevention and Travel Medicine, HOCH Health Ostschweiz, Cantonal Hospital St. Gallen, St. Gallen, Switzerland; b School of Medicine, University of St. Gallen, St. Gallen, Switzerland; c Children’s Hospital of Eastern Switzerland, St. Gallen, Switzerland; d Swiss National Reference Center for Invasive Pneumococci (NZPn), Institute for Infectious Diseases, University of Bern, Bern, Switzerland; e Institute for Infectious Diseases, University of Bern, Bern, Switzerland; f Lung Center, HOCH Health Ostschweiz, Cantonal Hospital St. Gallen, St. Gallen, Switzerland

**Keywords:** Invasive pneumococcal disease, incidence, pneumococcal serotypes, PCV-13, COVID-19

## Abstract

In Switzerland, thirteen-valent pneumococcal conjugate vaccine (PCV13) has been introduced in 2011. During the COVID-19 pandemic, cases of invasive pneumococcal disease (IPD) have decreased but consequences on the serotype epidemiology are less clear. The objective of the study has been to analyse the impact of PCV13 introduction and the COVID-19 pandemic on the IPD epidemiology and investigate the changes in the case fatality risk (CFR). We analysed data from the Swiss nationwide surveillance for the period 2012–2022. Poisson and logistic regression analyses were performed allowing us to inspect trends over time and to define serotypes that are associated with case fatality. In total, 8747 IPD cases were included from 2012 to 2022. IPD incidence dropped in the years 2020 (6.0/100,000) and 2021 (5.5/100,000) but recovered in 2022 (9.1/100,000). While the incidence numbers of patients >65 years did not reach the pre-pandemic level, numbers significantly increased in infants <1 year in 2022 (IRR 1.08, 95%CI: 1.01–1.16). The incidence of PCV13 serotypes among all IPD cases decreased until 2019 before increasing again during the pandemic (in 2022). Logistic regression analyses revealed that the PCV20 serotype 11A (OR: 1.76, 95%CI: 1.14–2.64), and the PCV13 serotypes 3 (OR: 1.26, 95% CI: 1.04–1.53) and 19F (OR: 1.76, 95%CI: 1.14–2.65) were significantly associated with increased CFR. In conclusion, the COVID-19 pandemic has had only minor temporary effects on the serotype distribution. Continued use of vaccines with extended serotype coverage may further reduce IPD disease burden and mortality.

## Introduction

*Streptococcus pneumoniae* (pneumococcus) causes severe clinical manifestations, including invasive pneumococcal disease (IPD) with a 30-day case fatality risk (CFR) of 8–30% [1–3]. Pneumococcal conjugate vaccine (PCV) programmes have been introduced globally and, as a consequence, the incidence of IPD dropped in many countries and remained fairly constant thereafter. This has been exemplified in an observational study from England and Wales on the effect of the 13-valent PCV (PCV13) on IPD four years after its introduction showing declines in all ages [4]. In Israel, four years after PCV7 and 2.5 years after PCV13 universal implementation in children, the incidence of adult IPD decreased [5]. In Denmark, a 21% reduction in IPD incidence in the total population after PCV13 introduction and a 28% decrease in IPD-related mortality have been observed [6]. IPD incidences in older adults in Denmark and Norway showed on average an annual decline from 2010 to 2019, whereas no change was seen for Sweden or Finland [7,8]. We reported a drop in incidence in adults 4 years after PCV7 introduction in Switzerland which was pronounced in older adults [9]. Despite low numbers, IPD incidences in children have generally also dropped and are now stable [10]. PCV13 was introduced in Switzerland in 2011 for children less than 5 years and, as a result, serotype replacement has been discovered [11].

Vaccination programmes had an impact on the serotype distribution and, as a consequence, non-vaccine serotypes have been increasing since then [4–6,10]. Initially, serotype replacement with non-PCV7 serotypes had been counteracted by the introduction of PCV13, but further replacement by non-PCV13 serotypes continues to diminish the overall vaccination effect [10,12]. In England and Wales, rapid increases in some non-PCV13 serotypes were observed with replacement, particularly in adult age groups [13]. Higher valent PCVs have recently been introduced or are ready to be launched. This includes PCV15 and/or PCV20 in children or adults. It is, therefore, important to especially monitor the disease burden due to PCV20-non-PCV15 or PCV15-non-PCV13 serotypes along with the introduction of these new programmes.

Apart from the vaccination programmes, the COVID-19 pandemic also had a major impact on the epidemiology of IPD. Laboratories from 26 countries and territories noted a significant and sustained reduction in IPD from 2018 to 2020 [14]. More specifically, COVID-19 containment measures were associated with a sustained decrease in the IPD incidence during the first 2 years of the pandemic, but cases began to increase as pandemic restrictions were lifted [15]. In Germany, IPD levels began to return to and even exceed seasonal levels in spring and summer 2021, following sharp declines in 2020 [16].

In this study, we hypothesized that there have been relevant changes in pneumococcal epidemiology after COVID-19 measures were loosened in the spring of 2021. A first, preliminary analysis without clinical information confirmed this assumption with the increase of serotype 23B [17]. In this current study, we aim to analyse temporal changes in pneumococcal serotype epidemiology from 2012 to 2022 combining clinical and microbiological data from invasive pneumococcal isolates.

## Material and methods

### Study design

IPD surveillance in Switzerland is characterized by a mandatory national laboratory-based surveillance system, which means that laboratories have to declare positive tests sampled from a sterile material and send their isolates to the national reference laboratory for typing. The physicians declare the clinical information after a positive laboratory test. Clinical information from IPD patients and the corresponding laboratory data are then combined at the Federal Office for Public Health (FOPH). The clinical data have been collected prospectively and clinical records were the source. Our dataset stems from the Swiss nationwide IPD database from 2012 to 2022, as provided by the FOPH. The data have been checked for duplicates. The IPD surveillance is part of the governmental public health surveillance based on the law for epidemics and is, therefore, exempted from approval by Institutional Review Boards.

### Data collection and processing

All data were anonymized. In this retrospective analysis, we focused on age, serotype, and death. We examined the proportions of different serotype groups and single serotypes, including those covered by the different pneumococcal conjugate vaccines (PCV13, PCV15, PCV20, and non-vaccine serotypes, NVT, i.e. not covered by PCV20). Mutually exclusive serotype groups were PCV13, PCV15-non-PCV13, PCV20-non-PCV15, and NVT. All serotypes of PCV20 were used in the single serotype analysis and explored alone with the exception of serotype 15B, which has been analysed together with 15C as 15B/C. NVT serotypes were examined individually if the overall proportion was above 0.5% for a serotype, otherwise grouped as other (non-typable, 10, 11, 12, 13, 15, 15F, 18, 18F, 19, 2, 21, 22, 25, 27, 28, 29, 33, 33A, 34, 35, 36, 37, 39, 41, 42, 6, 6D, and 9). Non-vaccine serotype groups 16 and 24 also were grouped, respectively, due to separate serotyping starting only in 2019 and were named 16/F (16, 16F) and 24/F (24, 24F). The serotype coverage of residual IPD for the currently used or planned vaccines was obtained (PCV13, PCV15, PCV20, and V116). The years of introduction of the vaccines in Switzerland can be appreciated in the supplementary figure. We also examined the frequency of different organ manifestations of IPD according to different age groups. Our dataset listed the following clinical manifestations: pneumonia, bacteremia without focus, meningitis, arthritis, and others. The first four were documented with either “yes” or an empty field, while “other” was documented with free text for the exact manifestation or left empty. Patients could have more than one manifestation. We, therefore, created the following mutually exclusive categories for this analysis: pneumonia (irrespective of other organ manifestations but without meningitis); meningitis (with or without other organ manifestations); bacteremia without focus; and other (i.e. not included in the first three groups). Crude mortality at the time of reporting by the clinician was also reported with either “yes” or an empty field, which could mean no death or unknown. Serotyping was performed by Quellung reaction using specific antisera from Statens Serum Institut.

### Data and statistical analysis

Incidence rates of IPD cases were calculated and stratified by age. Swiss population data were derived from the Federal Statistical Office. Poisson regression was used to analyse trends over time (2012-2022). The outcome of Poisson regression was reported using incidence rate ratios (IRR) and associated 95% CI. IRR correspond to the exponent of the Poisson regression coefficient. IRR provides the estimated rate ratio for a 1-unit (year) increase. An IRR > 1 indicates an average increase in the infection rate over time, whereas an IRR < 1 indicates an average decrease in the infection rate over time. A *p*-value <0.05 was considered statistically significant.

Multivariable logistic regression was conducted to test the association of death (outcome) with certain serotypes overall and in different age groups with no death of each serotype being the reference. Regression was adjusted by age and manifestation groups and used the same serotypes and serotype groups as in the trend analysis. Serotypes and groups had to cause more than 1 death to be included in the regression. The logistic regression estimates were expressed as odds ratio (OR) and 95% confidence interval (95% CI). All analyses were done using the R statistical software (v. 4.2.1).

## Results

### Incidence of invasive pneumococcal disease by demographic and clinical subgroups

Overall, 8747 IPD cases have been included during 2012-2022. The observed cases per 100,000 (overall incidence) remained relatively constant from 10.7/100,000 in 2012 to 9.6/100,000 in 2019 until the onset of COVID-19 when numbers decreased (Table 1). As of 2022, incidences were still lower (9.1/100,000) than those before the COVID-19 pandemic (IRR 0.96, 95%CI: 0.95–0.97). The incidences were higher in males than females but trends over time were comparable (Table 1). Age-stratified analyses revealed low absolute numbers in the paediatric age groups. However, incidence numbers in infants <1 year significantly increased after the COVID-19 pandemic (18.1/100,000 in 2021 and 19.8/100,000 in 2022) (IRR 1.08, 95%CI: 1.02–1.16). In the older adults aged 65 years or more, the IPD incidence also increased to 29.3/100,000 in 2022 but has not yet achieved pre-pandemic levels (32.5/100,000 in 2019) and, therefore, showed a decreased trend overall (IRR 0.95, 95%CI: 0.95–0.96). Deaths associated with IPD decreased during 2012-2019 (Table 1), further declined during the pandemic and remained lower in 2022 than those in 2019.

**Table 1.** Characteristics of the study population with invasive pneumococcal disease, Switzerland 2012-2022.

No. of cases per 100,000 population, no. of cases (%)												
	Year											IRR (95% CI, p-value)^a^
	2012	2013	2014	2015	2016	2017	2018	2019	2020	2021	2022	
Total	10.7	10.9	9.5	10.8	9.8	11.1	10.8	9.6	6.0	5.5	9.1	0.96 (0.95–0.97, <0.001)^b^
	861 (100)	891 (100)	786 (100)	900 (100)	821 (100)	943 (100)	920 (100)	826 (100)	518 (100)	481 (100)	800 (100)	
Female	9.8	10.5	8.8	10.1	9.0	10.1	10.0	8.8	4.8	4.3	7.9	0.95 (0.94–0.96, <0.001)^c^
	399 (46.3)	433 (48.6)	365 (46.4)	424 (47.1)	382 (46.5)	430 (45.6)	430 (46.7)	381 (46.1)	209 (40.3)	191 (39.7)	350 (43.8)	
Male	11.6	11.3	10.3	11.5	10.4	12.1	11.4	10.3	7.1	6.7	10.2	0.97 (0.96–0.98, <0.001)^c^
	461 (53.5)	455 (51.1)	418 (53.2)	475 (52.8)	434 (52.9)	510 (54.1)	485 (52.7)	439 (53.1)	304 (58.7)	290 (60.3)	448 (56.0)	
Death total	1.1	1.2	1.3	1.3	1.0	1.2	0.9	0.8	0.6	0.4	0.6	0.92 (0.90–0.94, <0.001)^b^
	85 (9.9)	94 (10.5)	107 (13.6)	108 (12.0)	82 (10.0)	99 (10.5)	81 (8.8)	68 (8.2)	51 (9.8)	38 (7.9)	49 (6.1)	
*Age-group*^d^												
0	10.0	4.9	10.7	9.4	9.3	8.2	10.4	8.3	8.3	18.1	19.8	1.08 (1.02–1.16, 0.01)^c^
	8 (0.9)	4 (0.4)	9 (1.1)	8 (0.9)	8 (1.0)	7 (0.7)	9 (1.0)	7 (0.8)	7 (1.4)	16 (3.3)	16 (2.0)	
1–4	7.7	7.6	5.4	5.6	3.8	4.3	8.2	4.5	2.3	4.3	7.4	0.97 (0.92–1.01, 0.11)^c^
	25 (2.9)	25 (2.8)	18 (2.3)	19 (2.1)	13 (1.6)	15 (1.6)	29 (3.2)	16 (1.9)	8 (1.5)	15 (3.1)	26 (3.3)	
5–16	1.5	3.2	1.8	2.0	1.2	0.9	1.9	1.3	1.3	1.6	2.8	0.99 (0.95–1.03, 0.63)^c^
	15 (1.7)	31 (3.5)	18 (2.3)	20 (2.2)	12 (1.5)	9 (1.0)	19 (2.1)	13 (1.6)	13 (2.5)	17 (3.5)	30 (3.8)	
17–64	6.2	6.4	4.8	5.7	5.0	5.3	5.7	4.8	3.6	3.2	4.1	0.95 (0.94–0.96, <0.001)^c^
	325 (37.7)	341 (38.3)	259 (33.0)	307 (34.1)	274 (33.4)	294 (31.2)	312 (33.9)	268 (32.4)	199 (38.4)	177 (36.8)	232 (29.0)	
65+	34.9	34.2	32.9	36.5	33.7	39.9	34.9	32.5	17.9	15.3	29.3	0.95 (0.95–096, <0.001)^c^
	488 (56.7)	490 (55.0)	482 (61.3)	546 (60.7)	514 (62.6)	618 (65.5)	550 (59.8)	522 (63.2)	291 (56.2)	255 (53.0)	495 (61.9)	
*Manifestation-group*												
Known	835 (97.0)	859 (96.4)	757 (96.3)	867 (96.3)	759 (92.4)	899 (95.3)	876 (95.2)	773 (93.6)	450 (86.9)	385 (80.0)	684 (85.5)	
Pneumonia (without Meningitis)	7.8	7.9	6.5	7.6	6.5	7.7	7.9	6.7	3.6	3.2	5.6	0.95 (0.94–0.95, <0.001)^b^
	629 (75.3)	642 (74.7)	533 (70.4)	637 (73.5)	544 (71.7)	657 (73.1)	671 (76.6)	578 (74.8)	311 (69.1)	276 (71.7)	496 (72.5)	
Meningitis	0.4	0.6	0.6	0.7	0.7	0.8	0.6	0.7	0.3	0.3	0.7	0.98 (0.95–1.01, 0.2)^b^
	36 (4.3)	47 (5.5)	50 (6.6)	61 (7.0)	60 (7.9)	65 (7.2)	47 (5.4)	57 (7.4)	29 (6.4)	24 (6.2)	60 (8.8)	
Bacteremia without focus	1.4	1.4	1.3	1.4	1.1	1.0	1.1	0.9	0.7	0.5	0.6	0.91 (0.89–0.93, <0.001)^b^
	109 (13.1)	116 (13.5)	104 (13.7)	114 (13.1)	90 (11.9)	87 (9.7)	95 (10.8)	76 (9.8)	61 (13.6)	40 (10.4)	50 (7.3)	
Other	0.8	0.7	0.8	0.7	0.8	1.1	0.7	0.7	0.6	0.5	0.9	0.99 (0.97–1.01, 0.4)^b^
	61 (7.3)	54 (6.3)	70 (9.2)	55 (6.3)	65 (8.6)	90 (10.0)	63 (7.2)	62 (8.0)	49 (10.9)	45 (11.7)	78 (11.4)	

^a^ Poisson Regression; ^b^N = population; ^c^N = group-stratified population;^d^three times age not known.

The most frequent IPD manifestation was pneumonia followed by bacteremia without focus and meningitis (Table 1). In this case, bacteraemia secondary to/concurrent with pneumonia was classified under pneumonia. After the COVID-19 pandemic, incidences of meningitis and “other” manifestations again reached 0.7/100,000 and 0.9/100,000, which was as high or higher than observed in 2019 and responsible for a higher percentage of IPD than bacteremia without a focus in 2022. In contrast, incidences of pneumonia and bacteremia without a focus have not achieved pre-pandemic 2019 values (5.6 vs. 6.7/100,000 for pneumonia and 0.6 vs. 0.9/100,000 for bacteremia without focus) yet. The distribution of the focus of infection was similar for the different age groups (Supplementary Table 1).

We received 935 blood and 34 “other” samples from 942 patients with bacteremia without a focus. As for patients with pneumonia without meningitis, 5903 blood, 85 pleural fluid, and 56 “other” samples were received. As for patients with meningitis 419 blood, 319 CSF, and 13 “other” samples were received. For patients with “other” manifestations, the majority of samples were from blood (*n* = 576) and synovial fluid (*n* = 63).

### Serotype-groups distribution of IPD in Switzerland

**Figure 1.**
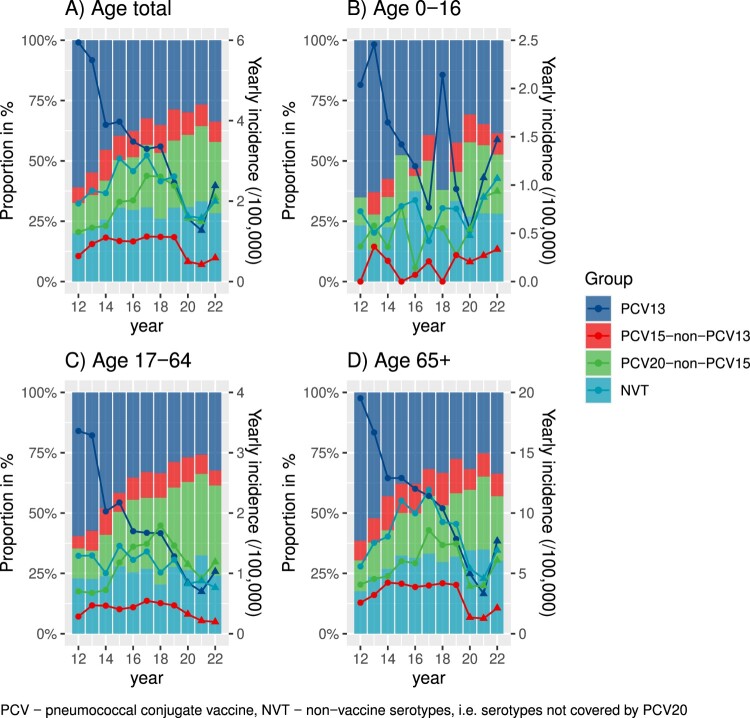
Incidence and proportions of serotype–groups causing invasive pneumococcal disease over time. Age group-stratified proportions of serotype groups PCV13, PCV15-13, PCV20-15, and NVT are shown overall as bar plots (A), for patients aged 0–16 (B), 17–64 (C) and older than 65 years (D); The connected dots indicate the incidence of IPD cases. PCV – pneumococcal conjugate vaccine, NVT – non-vaccine serotypes, i.e. serotypes not covered by PCV20.

We next assessed the incidence and proportions by serotype groups over time (Figure 1 and Supplementary Table 2). We found that the proportion of PCV13 serotypes decreased from 61.0% in 2012 to 28.7% in 2019, and slightly increased in 2022 (33.8%) compared to 2020–21 (Poisson Regression; *p* < 0.001; Figure 1(A)). In contrast, the proportion of PCV15-non-PCV13 slightly increased and then remained constant over the study period (6.5% in 2012, 11% in 2017, 8.4% in 2022, *p* = 0.3). The proportion of PCV20-non-PCV15 group IPD significantly increased from 2012 to 2019 with similar values in 2022 (12.6% in 2012, 27.9% in 2019, 29.6% in 2022, *p* < 0.001). Finally, the proportion of NVT increased from 2012 to 2019 and remained constant thereafter (19.9% in 2012, 30.6% in 2019, 28.3% in 2022, *p* < 0.001).

We then stratified the incidence numbers with different serotypes according to three age groups (Figure 1(B–D); Supplementary Table 2). In the patients aged 0–16 years (Figure 1(B)), cases of IPD due to PCV13 serotypes decreased until 2019 and remained low during the pandemic, but again increased in 2022 (*n* = 22 cases) compared to 2019 (*n* = 14). Cases with PCV15-non-PCV13, PCV20-non-PCV15 and NVT serotypes have been increasing since 2012, illustrating the above-mentioned finding of a general increase of IPD cases in the young age group.

As for the two age groups 17–64 years and 65+ years of age, the incidence numbers in all serotype groups were still lower in 2022 than 2019 (Figure 1(C,D)). More specifically, PCV15-non-PCV13 dipped in proportion during and after the pandemic after a consistent increase from 2012 to –2019 but did not yet fully recover in 2022 (Supplementary Table 2). The PCV20-non-PCV15 serotypes were responsible for a considerable burden of IPD in all age groups in Switzerland in 2022. Grouping the serotypes according to planned or already used vaccines,shows that serotypes in PCV20 and particularly V116 are responsible for the majority of IPD in children and adults (Supplementary Table 3).

### Single serotype distribution

As for single-serotype distribution, PCV13 vaccine serotypes have markedly decreased with the exception of serotype 3 (*n* = 137 in 2022) (Table 2). Nevertheless, compared to pre-pandemic year 2019, higher numbers of 19F were noted in 2022 (Poisson regression; *p* < 0.02). For the single serotypes of the higher-valent PCVs, numbers of serotype 22F were still high in 2022 but did not reach pre-pandemic levels yet. Serotype 8 has remained together with serotype 3 the leading serotype since 2019. Single serotype analyses of non-PCV20 serotypes still showed generally low numbers, with 15A, 23B, 7, and 9N generally increasing in proportion (Poisson regression; *p* < 0.001). Overall, serotypes 23B and 9N were the most frequent non-PCV20 serotypes. Due to the high numbers of IPD in the 65+ group, the overall single serotype analyses partly and closely reflect the situation in the 17–64 and 65+ group, respectively. (Supplementary Tables 4 and 5).

**Table 2.** Serotypes of Streptococcus pneumoniae causing invasive pneumococcal disease (IPD) across ages, Switzerland 2012-2022.

No. of cases per 100,000 population, IPD-cases with serotype known no. (%)												
Serotype	Year											IRR (95% CI, p-value)^a^
	2012	2013	2014	2015	2016	2017	2018	2019	2020	2021	2022	
Known	784 (91.1)	817 (91.7)	706 (89.8)	835 (92.8)	778 (94.8)	864 (91.6)	818 (88.9)	736 (89.1)	456 (88)	416 (86.5)	622 (77.8)	
*PCV13*^b^												
1	0.36	0.28	0.24	0.07	0.05	0.04	0.00	0.01	0.00	0.00	0.00	0.58 (0.51–0.65, <0.001)^c^
	29 (3.7)	23 (2.8)	20 (2.8)	6 (0.7)	4 (0.5)	3 (0.3)	0 (0,0)	1 (0.1)	0 (0.0)	0 (0.0)	0 (0.0)	0.59 (0.52–0.66, <0.001)^d^
3	1.49	1.68	1.34	1.66	1.78	1.59	2.01	1.39	0.82	0.80	1.55	0.97 (0.95–0.98, <0.001)^c^
	120 (15.3)	137 (16.8)	110 (15.6)	138 (16.5)	150 (19.3)	135 (15.6)	172 (21.0)	120 (16.3)	71 (15.6)	70 (16.8)	137 (22.0)	1.02 (1.00–1.04, 0.02)^d^
4	0.31	0.28	0.22	0.22	0.21	0.22	0.14	0.08	0.07	0.07	0.12	0.87 (0.83–0.92, <0.001)^c^
	25 (3.2)	23 (2.8)	18 (2.5)	18 (2.2)	18 (2.3)	19 (2.2)	12 (1.5)	7 (1.0)	6 (1.3)	6 (1.4)	11 (1.8)	0.91 (0.86–0.96, <0.001)^d^
5	0.02	0.05	0.04	0.01	0.00	0.00	0.00	0.00	0.00	0.00	0.00	0.56 (0.36–0.77, 0.002)^c^
	2 (0.3)	4 (0.5)	3 (0.4)	1 (0.1)	0 (0.0)	0 (0.0)	0 (0.0)	0 (0.0)	0 (0.0)	0 (0.0)	0 (0.0)	0.57 (0.37–0.79, 0.004)^d^
6A	0.26	0.17	0.21	0.13	0.07	0.06	0.06	0.01	0.03	0.00	0.01	0.73 (0.67–0.79, <0.001)^c^
	21 (2.7)	14 (1.7)	17 (2.4)	11 (1.3)	6 (0.8)	5 (0.6)	5 (0.6)	1 (0.1)	3 (0.7)	0 (0.0)	1 (0.2)	0.75 (0.68–0.82, <0.001)^d^
6B	0.12	0.06	0.08	0.02	0.06	0.11	0.02	0.03	0.05	0.01	0.02	0.86 (0.79–0.95, 0.002)^c^
	10 (1.3)	5 (0.6)	7 (1.0)	2 (0.2)	5 (0.6)	9 (1.0)	2 (0.2)	3 (0.4)	4 (0.9)	1 (0.2)	2 (0.3)	0.90 (0.82–0.99, 0.04)^d^
7F	1.09	0.96	0.45	0.43	0.27	0.18	0.09	0.06	0.03	0.01	0.03	0.67 (0.63–0.70, <0.001)^c^
	88 (11.2)	78 (9.5)	37 (5.2)	36 (4.3)	23 (3.0)	15 (1.7)	8 (1.0)	5 (0.7)	3 (0.7)	1 (0.2)	3 (0.5)	0.69 (0.65–0.72, <0.001)^d^
9V	0.14	0.22	0.05	0.07	0.07	0.08	0.08	0.12	0.00	0.00	0.02	0.83 (0.76–0.90, <0.001)^c^
	11 (1.4)	18 (2.2)	4 (0.6)	6 (0.7)	6 (0.8)	7 (0.8)	7 (0.9)	10 (1.4)	0 (0.0)	0 (0.0)	2 (0.3)	0.86 (0.79–0.94, <0.001)^d^
14	0.35	0.47	0.36	0.35	0.19	0.19	0.21	0.21	0.14	0.02	0.02	0.83 (0.79–0.87, <0.001)^c^
	28 (3.6)	38 (4.7)	30 (4.2)	29 (3.5)	16 (2.1)	16 (1.9)	18 (2.2)	18 (2.4)	12 (2.6)	2 (0.5)	2 (0.3)	0.86 (0.82–0.91, <0.001)^d^
18C	0.14	0.09	0.13	0.05	0.05	0.01	0.06	0.09	0.05	0.03	0.01	0.86 (0.79–0.94, <0.001)^c^
	11 (1.4)	7 (0.9)	11 (1.6)	4 (0.5)	4 (0.5)	1 (0.1)	5 (0.6)	8 (1.1)	4 (0.9)	3 (0.7)	1 (0.2)	0.90 (0.82–0.98, 0.02)^d^
19A	1.18	0.81	0.51	0.72	0.48	0.58	0.41	0.22	0.25	0.16	0.27	0.84 (0.82–0.87, <0.001)^c^
	95 (12.1)	66 (8.1)	42 (5.9)	60 (7.2)	40 (5.1)	49 (5.7)	35 (4.3)	19 (2.6)	22 (4.8)	14 (3.4)	24 (3.9)	0.88 (0.85–0.91, <0.001)^d^
19F	0.14	0.27	0.19	0.14	0.21	0.21	0.22	0.20	0.09	0.15	0.28	1.00 (0.96–1.05, 0.90)^c^
	11 (1.4)	22 (2.7)	16 (2.3)	12 (1.4)	18 (2.3)	18 (2.1)	19 (2.3)	17 (2.3)	8 (1.8)	13 (3.1)	25 (4.0)	1.06 (1.01–1.11, 0.02)^d^
23F	0.34	0.16	0.07	0.10	0.04	0.04	0.05	0.02	0.03	0.01	0.02	0.72 (0.65–0.79, <0.001)^c^
	27 (3.4)	13 (1.6)	6 (0.8)	8 (1.0)	3 (0.4)	3 (0.3)	4 (0.5)	2 (0.3)	3 (0.7)	1 (0.2)	2 (0.3)	0.74 (0.67–0.82, <0.001)^d^
*PCV15-non-PCV13*												
22F	0.61	0.88	0.95	0.84	0.88	0.92	0.98	0.96	0.42	0.34	0.52	0.95 (0.93–0.98, <0.001)^c^
	49 (6.2)	72 (8.8)	78 (11.0)	70 (8.4)	74 (9.5)	78 (9.0)	84 (10.3)	83 (11.3)	36 (7.9)	30 (7.2)	46 (7.4)	1.00 (0.98–1.03, 0.77)^d^
33F	0.02	0.05	0.15	0.17	0.12	0.20	0.13	0.14	0.08	0.08	0.07	1.00 (0.94–1.07, 0.88)^c^
	2 (0.3)	4 (0.5)	12 (1.7)	14 (1.7)	10 (1.3)	17 (2.0)	11 (1.3)	12 (1.6)	7 (1.5)	7 (1.7)	6 (1.0)	1.06 (1.00–1.13, 0.06)^d^
*PCV20-non-PCV15*												
8	0.57	0.66	0.57	1.00	1.19	1.53	1.56	1.31	0.93	1.03	1.54	1.07 (1.05–1.09, <0.001)^c^
	46 (5.9)	54 (6.6)	47 (6.7)	83 (9.9)	100 (12.9)	130 (15.0)	133 (16.3)	113 (15.4)	81 (17.8)	90 (21.6)	136 (21.9)	1.14 (1.12–1.16, <0.001)^d^
10A	0.16	0.18	0.22	0.25	0.21	0.29	0.21	0.26	0.17	0.15	0.17	0.99 (0.95–1.03, 0.61)^c^
	13 (1.7)	15 (1.8)	18 (2.5)	21 (2.5)	18 (2.3)	25 (2.9)	18 (2.2)	22 (3.0)	15 (3.3)	13 (3.1)	15 (2.4)	1.04 (1.00–1.09, 0.07)^d^
11A	0.17	0.20	0.22	0.25	0.23	0.26	0.20	0.17	0.14	0.15	0.12	0.96 (0.91–1.00, 0.08)^c^
	14 (1.8)	16 (2.0)	18 (2.5)	21 (2.5)	19 (2.4)	22 (2.5)	17 (2.1)	15 (2.0)	12 (2.6)	13 (3.1)	11 (1.8)	1.01 (0.96–1.06, 0.68)^d^
12F	0.15	0.17	0.18	0.28	0.18	0.28	0.42	0.48	0.12	0.06	0.10	0.99 (0.94–1.03, 0.55)^c^
	12 (1.5)	14 (1.7)	15 (2.1)	23 (2.8)	15 (1.9)	24 (2.8)	36 (4.4)	41 (5.6)	10 (2.2)	5 (1.2)	9 (1.4)	1.04 (1.00–1.09, 0.07)^d^
15B/C^e^	0.17	0.12	0.19	0.20	0.21	0.26	0.22	0.16	0.21	0.10	0.15	0.99 (0.94–1.03, 0.58)^c^
	14 (1.8)	10 (1.2)	16 (2.3)	17 (2.0)	18 (2.3)	22 (2.5)	19 (2.3)	14 (1.9)	18 (3.9)	9 (2.2)	13 (2.1)	1.04 (0.99–1.09, 0.11)^d^
*NVT*^f^												
10B	0.04	0.02	0.04	0.06	0.05	0.05	0.05	0.05	0.03	0.06	0.05	1.03 (0.93–1.13, 0.58)^c^
	3 (0.4)	2 (0.2)	3 (0.4)	5 (0.6)	4 (0.5)	4 (0.5)	4 (0.5)	4 (0.5)	3 (0.7)	5 (1.2)	4 (0.6)	1.09 (0.98–1.21, 0.10)^d^
15A	0.07	0.10	0.21	0.28	0.25	0.32	0.25	0.30	0.12	0.10	0.22	1.02 (0.97–1.07, 0.39)^c^
	6 (0.8)	8 (1.0)	17 (2.4)	23 (2.8)	21 (2.7)	27 (3.1)	21 (2.6)	26 (3.5)	10 (2.2)	9 (2.2)	19 (3.1)	1.08 (1.03–1.13, 0.001)^d^
16/F^e^	0.05	0.01	0.06	0.07	0.13	0.17	0.11	0.10	0.09	0.05	0.08	1.04 (0.97–1.12, 0.24)^c^
	4 (0.5)	1 (0.1)	5 (0.7)	6 (0.7)	11 (1.4)	14 (1.6)	9 (1.1)	9 (1.2)	8 (1.8)	4 (1.0)	7 (1.1)	1.11 (1.03–1.19, 0.007)^d^
17F	0.07	0.10	0.06	0.08	0.10	0.05	0.08	0.10	0.00	0.07	0.07	0.96 (0.89–1.04, 0.31)^c^
	6 (0.8)	8 (1.0)	5 (0.7)	7 (0.8)	8 (1.0)	4 (0.5)	7 (0.9)	9 (1.2)	0 (0.0)	6 (1.4)	6 (1.0)	1.01 (0.94–1.10, 0.75)^d^
20	0.04	0.07	0.06	0.23	0.08	0.24	0.09	0.09	0.02	0.03	0.05	0.95 (0.89–1.01, 0.13)^c^
	3 (0.4)	6 (0.7)	5 (0.7)	19 (2.3)	7 (0.9)	20 (2.3)	8 (1.0)	8 (1.1)	2 (0.4)	3 (0.7)	4 (0.6)	1.00 (0.93–1.07, 0.97)^d^
23A	0.10	0.18	0.25	0.20	0.15	0.26	0.22	0.30	0.12	0.13	0.11	0.98 (0.94–1.03, 0.47)^c^
	8 (1.0)	15 (1.8)	21 (3.0)	17 (2.0)	13 (1.7)	22 (2.5)	19 (2.3)	26 (3.5)	10 (2.2)	11 (2.6)	10 (1.6)	1.04 (0.99–1.09, 0.15)^d^
23B	0.14	0.16	0.22	0.32	0.20	0.35	0.30	0.19	0.17	0.30	0.25	1.03 (0.99–1.07, 0.16)^c^
	11 (1.4)	13 (1.6)	18 (2.5)	27 (3.2)	17 (2.2)	30 (3.5)	26 (3.2)	16 (2.2)	15 (3.3)	26 (6.2)	22 (3.5)	1.09 (1.04–1.14, <0.001)^d^
24/F^e^	0.11	0.20	0.22	0.42	0.31	0.15	0.13	0.15	0.10	0.07	0.11	0.92 (0.88–0.97, <0.001)^c^
	9 (1.1)	16 (2.0)	18 (2.5)	35 (4.2)	26 (3.3)	13 (1.5)	11 (1.3)	13 (1.8)	9 (2.0)	6 (1.4)	10 (1.6)	0.97 (0.92–1.02, 0.20)^d^
31	0.07	0.02	0.07	0.12	0.14	0.15	0.06	0.07	0.05	0.03	0.03	0.95 (0.88–1.02, 0.15)^c^
	6 (0.8)	2 (0.2)	6 (0.8)	10 (1.2)	12 (1.5)	13 (1.5)	5 (0.6)	6 (0.8)	4 (0.9)	3 (0.7)	3 (0.5)	1.00 (0.92–1.08, 0.93)^d^
35B	0.06	0.01	0.04	0.11	0.05	0.14	0.09	0.06	0.10	0.07	0.05	1.04 (0.96–1.12, 0.37)^c^
	5 (0.6)	1 (0.1)	3 (0.4)	9 (1.1)	4 (0.5)	12 (1.4)	8 (1.0)	5 (0.7)	9 (2.0)	6 (1.4)	4 (0.6)	1.10 (1.01–1.19, 0.02)^d^
35F	0.15	0.14	0.13	0.22	0.15	0.15	0.14	0.19	0.13	0.13	0.19	1.00 (0.95–1.06, 0.85)^c^
	12 (1.5)	11 (1.3)	11 (1.6)	18 (2.2)	13 (1.7)	13 (1.5)	12 (1.5)	16 (2.2)	11 (2.4)	11 (2.6)	17 (2.7)	1.06 (1.01–1.12, 0.03)^d^
38	0.07	0.11	0.12	0.10	0.12	0.06	0.07	0.08	0.07	0.00	0.03	0.90 (0.83–0.97, 0.005)^c^
	6 (0.8)	9 (1.1)	10 (1.4)	8 (1.0)	10 (1.3)	5 (0.6)	6 (0.7)	7 (1.0)	6 (1.3)	0 (0.0)	3 (0.5)	0.94 (0.87–1.02, 0.12)^d^
6C	0.21	0.43	0.12	0.28	0.20	0.22	0.16	0.13	0.10	0.09	0.06	0.88 (0.83–0.92, <0.001)^c^
	17 (2.2)	35 (4.3)	10 (1.4)	23 (2.8)	17 (2.2)	19 (2.2)	14 (1.7)	11 (1.5)	9 (2.0)	8 (1.9)	5 (0.8)	0.92 (0.87–0.97, 0.001)^d^
7	0.02	0.06	0.00	0.01	0.01	0.01	0.06	0.06	0.06	0.05	0.11	1.18 (1.06–1.32, 0.003)^c^
	2 (0.3)	5 (0.6)	0 (0.0)	1 (0.1)	1 (0.1)	1 (0.1)	5 (0.6)	5 (0.7)	5 (1.1)	4 (1.0)	10 (1.6)	1.26 (1.13–1.41, <0.001)^d^
9N	0.39	0.31	0.30	0.41	0.63	0.66	0.48	0.63	0.33	0.31	0.36	1.00 (0.97–1.04, 0.79)^c^
	31 (4.0)	25 (3.1)	25 (3.5)	34 (4.1)	53 (6.8)	56 (6.5)	41 (5.0)	54 (7.3)	29 (6.4)	27 (6.5)	32 (5.1)	1.06 (1.03–1.10, <0.001)^d^
Other^g^	0.34	0.33	0.29	0.16	0.17	0.15	0.20	0.12	0.13	0.10	0.23	0.91 (0.87–0.96 <0.001)^c^
	27 (3.4)	27 (3.3)	24 (3.4)	13 (1.6)	14 (1.8)	13 (1.5)	17 (2.1)	10 (1.4)	11 (2.4)	9 (2.2)	20 (3.2)	0.96 (0.91–1.01, 0.09)^d^

^a^ Poisson Regression.

^b^ Pneumococcal conjugate vaccine.

^c^ N = population.

^d^ N = total number of cases with serotype known.

^e^ 15B/C = 15B, 15C. 16/F = 16, 16F. 24/F = 24, 24F.

^f^ Non-vaccine serotypes, i.e. not covered by PCV20.

^g^ Non-vaccine serotypes with overall proportion <0.5% combined to other: non-typable, 10, 11, 12, 13, 15, 15F, 18, 18F, 19, 2, 21, 22, 25, 27, 28, 29, 33, 33A, 34, 35, 36, 37, 39, 41, 42, 6, 6D, and 9.

### Serotype association with death

The number of deaths in the 0–16 age group was very low resulting in low CFRs (Table 3). In contrast, the highest number of deaths and CFRs were found in the 65+ age group. Serotype 3 had the highest number of deaths (*n* = 148, CFR = 10.9%). Vaccine serotypes 3, 14, 19F, 11A, and non-vaccine serotypes 6C, 24/F, and 31 had high CFRs (Table 3). However, due to low case numbers for the majority of the remaining serotypes, the uncertainty of the CFR is large.

**Table 3.** Serotypes causing death in invasive pneumococcal disease (IPD) across ages, Switzerland 2012–2022.

IPD-cases with serotypes and outcome death known, no., CFR in % (95% CI)					
Serotype	Age-group				
	Total		Age 0–16	Age 17–64	Age 65+
	No.	CFR in % (95% CI)	No.	No.	No.
*PCV13*^a^					
3	148	10.9 (9.3–12.7)	2	30	116
4	15	9.2 (5.4–15.0)	0	5	10
5	2	20.0 (3.5–55.8)	0	1	1
6A	7	8.3 (3.7–17.0)	0	1	6
6B	6	12.0 (5.0–25.0)	0	0	6
7F	19	6.4 (4.0–10.0)	0	1	18
9V	10	14.1 (7.3–24.8)	0	2	8
14	31	14.8 (10.4–20.5)	0	2	29
18C	8	13.6 (6.4–25.5)	0	4	4
19A	45	9.7 (7.2–12.8)	0	9	36
19F	29	16.2 (11.3–22.6)	0	5	24
23F	6	8.3 (3.4–17.9)	0	4	2
*PCV15-non-PCV13*					
22F	57	8.1 (6.3–10.5)	1	15	41
33F	13	12.7 (7.2–21.2)	0	2	11
*PCV20-non-PCV15*					
8	43	4.2 (3.5–5.7)	0	10	33
10A	24	12.4 (8.3–18.1)	0	6	18
11A	29	16.3 (11.4–22.7)	0	5	24
12F	14	6.9 (3.9–11.5)	0	6	8
15B/C^b^	15	8.8 (5.2–14.4)	1	4	10
*NVT*^c^					
10B	1	2.4 (0.1–14.4)	0	0	1
15A	14	7.5 (4.3–12.5)	1	1	12
16/F^b^	11	14.1 (7.6–24.3)	0	2	9
17F	9	13.6 (6.8–24.8)	0	1	8
20	9	10.6 (5.3–19.6)	0	3	6
23A	17	9.9 (6.0–15.6)	0	2	15
23B	20	9.0 (5.8–13.8)	0	4	16
24/F^b^	22	13.3 (8.7–19.6)	2	7	13
31	13	18.6 (10.6–30.0)	0	2	11
35B	8	12.1 (5.7–23.0)	0	2	6
35F	18	12.4 (7.7–19.2)	0	4	14
38	3	4.3 (1.1–12.8)	0	0	3
6C	27	16.1 (11.0–22.7)	0	2	25
7	4	10.3 (3.3–25.2)	0	1	3
9N	33	8.1 (5.7–11.3)	1	6	26
Other^d^	23	12.4 (8.2–18.3)	1	9	13

^a^ Pneumococcal conjugate vaccine, serotype 1 did not cause any death.

^b^ 15B/C = 15B, 15C. 16/F = 16, 16F. 24/F = 24, 24F.

^c^ Non-vaccine serotypes, i.e. serotypes not covered by PCV20.

^d^ Non-vaccine serotypes with overall proportion <0.5% combined to other: non-typable, 10, 11, 12, 13, 15, 15F, 18, 18F, 19, 2, 21, 22, 25, 27, 28, 29, 33, 33A, 34, 35, 36, 37, 39, 41, 42, 6, 6D, and 9.

**Figure 2.**
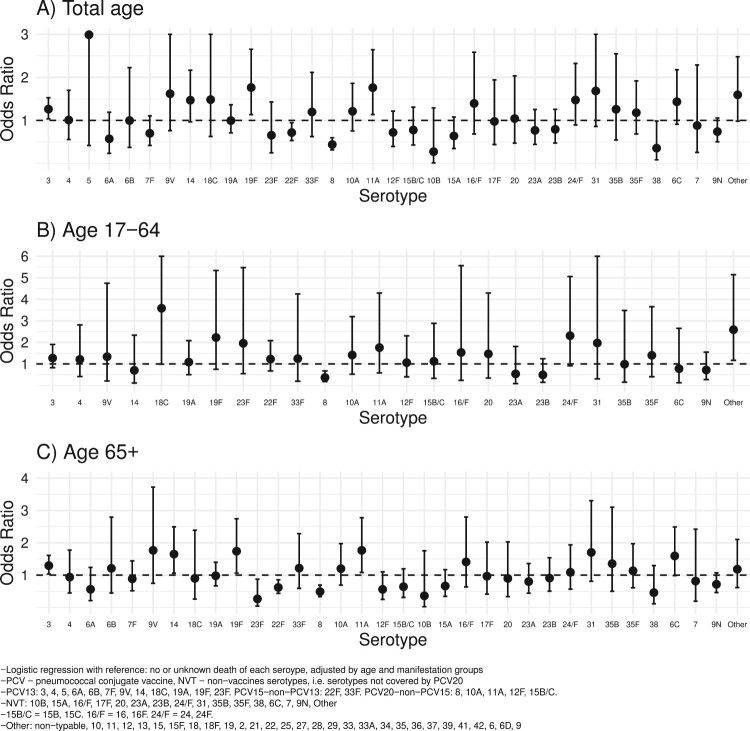
Association of death and serotypes/serogroups invasive pneumococcal disease across ages. Multivariable logistic regression analysis of serotype/serogroup associated with death. Odds ratios (and 95% CI) of selected serotypes are shown from the serotype groups PCV13, PCV15-13, PCV20-15, and NVT (A). In addition, the data are also shown in a sub-analysis for patients 17–64 (B) and >64 years of age (C); PCV – pneumococcal conjugate vaccine, NVT – non-vaccine serotypes, i.e. serotypes not covered by PCV20.

We next performed multivariable logistic regression analysis to investigate the association of the different serotypes or groups of serotypes with death. We found that the PCV13 group was positively associated with death (OR: 1.19, 95%CI: 1.03–1.39) while the opposite was true for the two other vaccine types groups (PCV15-non-PCV13: OR: 0.78, 95%CI: 0.59–1.0) (PCV20-non-PCV15: OR: 0.71, 95%CI: 0.58–0.86). There was no association between the non-PCV20 group and death (OR: 0.99, 95%CI: 0.84–1.17). As for individual vaccine serotypes, serotype 3, 11A and 19F were positively associated with death (OR and 95%CI >1.0), while serotype/serogroup 7F and 8 were associated with survival (OR and 95%CI <1.0) (Figure 2(A)). Similar trends were found for both the 17–64 and the 65+ age group, with the exception that serotype 18C had a higher effect in the 17–64 age group (OR: 3.58, 95%CI: 0.99–10.20) as compared to the 6+ age group (OR: 0.9, 95%CI: 0.26–2.39). However, these associations were statistically not significant due to the fewer cases. Serotypes 19F and 11A were more strongly associated with death in the age group 65+ (19F: OR: 1.74, 95%CI: 1.06–2.75; 11A: OR: 1.77, 95%CI: 1.08–2.78) than the 17–64 age group (19F: OR: 2.23, 95%CI: 0.75–5.34, 11A: OR: 1.76, 95%CI: 0.58–4.29), although the associations for the age group 17–64 were statistically not significant (Figure 2(B,C)).

None of the non-vaccine serotypes was significantly associated with death but there was a trend for the serotypes 6C, 24/F, 31, and “other” as they had the highest OR values (Figure 2(A)). Stratifying for the two age groups, no significant OR was again noted (Figure 2(B,C)).

## Discussion

Our study reveals important findings regarding the epidemiology of IPD and the distribution and severity of serotypes causing IPD. The effects of the COVID-19 pandemic on IPD incidence were temporary with a recovery by 2022. The rebound was fastest in the paediatric age groups and slowest in the elderly. The proportion of PCV13 serotypes was slightly higher during the pandemic than immediately before. However, the proportion of IPD caused by serotypes included in higher-valency PCVs has been steadily increasing over time. Finally, we noted that CFRs varied largely by serotypes and tended to be higher for PCV13 serotypes than others.

IPD rates have generally decreased in most countries following the national PCV introduction [18]. This includes both the incidences in the paediatric and the adult population due to an indirect effect which may depend on national vaccination coverage rates [19–21]. However, replacement by non-vaccine serotypes remains a problem for IPD and after an initial decline, the overall IPD incidence seems to have reached a plateau at levels higher than desired [22,23]. Our Swiss national data mirror these developments with additional but temporary reductions during the first 2 COVID-19 pandemic years. The rebound was particularly apparent for the youngest age groups with a lag in the elderly. In addition, PCV13 serotypes were associated with high CFRs as already shown in our study using data from 2003 to 2012 [9] while there were no uniform trends among replacement serotypes with some showing high (e.g. 6C 24/F and 31) and others low (e.g. 7F and 8) CFRs. Different serotypes have been associated with CFRs in various publications. In particular, 3, 19F, and 24F have been associated with high case fatality, and serotypes 7F and 8 with a low case fatality [24–29]. Recent projects, which involve whole genome sequencing of pneumococcal isolates, have contributed to our understanding if increased case fatality is due to the serotype per se or the genomic background as certain clones are more virulent than others [30]. Overall, the introduction of higher-valent vaccines presents an opportunity to further mitigate serotype replacement and clinical IPD severity. The lower number of deaths since the COVID-19 pandemic may be best explained by lower incidences of IPD in the elderly in 2022 than in 2019.

Several studies have reported recent serotype redistribution and the upcoming non-vaccine serotypes in IPD [10,12,13,31,32]. In our as in other studies, a particular increase in serotype 8 and 22F was seen while the PCV13 serotype 3 remained high [10,13,33]. During the COVID-19 pandemic, there was a significant reduction in the risk of pneumococcal disease but no major changes in the distribution of cases were observed by patient age or serotype or group as data from Israel and Portugal suggest [15,34,35]. However, it is important to note that information is only slowly emerging on how the IPD incidence numbers and epidemiology have changed after the pandemic measures had been lifted. One of the few studies showed that IPD levels began to return to and exceed seasonal levels, for example, in spring and summer 2021 in Germany [16]. In Spain, IPD cases had returned to pre-pandemic levels in children, and partially in adults by 2022 and, therefore, very much mirror the results of our study [36]. Our data also indicate that no major serotype shifts occurred during the COVID-19 pandemic beyond secular trends. Similarly, the proportions of vaccine serotypes remained largely consistent throughout 2020-2021 in Germany [16]. Therefore, emerging PCV15-non-PCV13 and PCV20-non-PCV15 serotypes cause a substantial burden of the disease also after the COVID-19 pandemic, justifying the introduction of PCV15 and/or PCV20. In our study, additional serotypes not covered by PCV13 significantly increased and the introduction of PCV15 and particularly PCV20 are expected to have a great impact on the serotype distribution and IPD incidence in Switzerland, as it has in other settings [37]. In addition, V116 with a complementary serotype composition and 8 unique serotypes is expected to provide the highest disease coverage in adults.

However, apart from the disease burden, case fatality investigation is also important to consider when monitoring the effects of higher valent vaccine introduction. In France, it was found that 80% of the serotypes with high mortality potential were included in licensed PCV13 or PPV23 vaccines among 771 enrolled patients (with a median age of 66 years) [38]. In a 2010 systematic review and meta-analysis of serotype-specific disease outcomes for patients with pneumococcal pneumonia and meningitis [27], serotype-specific estimates of risk of death (risk ratio [RR]) were analysed. Overall, serotypes 1, 7F, and 8 were associated with decreased RRs, and serotypes 3, 6A, 6B, 9N, and 19F were associated with increased RRs. Relatively low CFRs were also found for serotypes 7F and 8 in our study. Serotypes 1 and 7F are often the most frequent in healthy adults and, therefore, this could explain their lower mortality rates. In contrast, the PCV13 serotypes 3 and 19F showed increased death rates as did the non-vaccine serotypes 11A, 6C, 24/F and 31. Therefore, in general, the serotypes which are associated with death are remarkably similar over time and in geographical settings despite the introduction of PCVs and pandemic changes. Unfortunately, the effectiveness of PCV13 against serotype 3 is controversial, so continued use of PCV13 may not further reduce the death rates [39].

A major strength of our study is the reporting of a major nationwide, population-based study which includes 8747 IPD cases. We estimate that we cover around 90% of IPD cases in the Swiss population being, therefore, representative of the whole country [9].

A limitation of our study is that around 10–15% of cases had missing information concerning clinical and/or serotype information. Missing data were related to individual laboratories and cantons providing less data which are scattered throughout the country. Therefore, this is unlikely to have introduced a bias in the main findings of our study. Despite overall high numbers, single serotype analyses are somewhat challenging for the less common serotypes resulting in large confidence intervals. However, their close monitoring is crucial as some of them, especially the non-vaccine serotypes are expected to rise in the near future. A limitation of the database is that the vaccination status of the analysed IPD cases is unknown. However, recent analyses have shown that vaccine uptake increased and reached >90% vaccine coverage in children (93% for 1 dose, 89% for 3 doses in 2020-2022; https://www.bag.admin.ch/dam/bag/de/dokumente/mt/i-und-b/durchimpfung/bu-13-24-durchimpfung-2020-2022.pdf.download.pdf/bu-13-24-durchimpfung-2020-2022-de.pdf) [11]. Therefore, it can be assumed that the vast majority of the paediatric population has been vaccinated. In contrast, adult vaccination rates are low in Switzerland. In 2020 a nationwide, cross-sectional survey of vaccination records to evaluate pneumococcal vaccination coverage and factors affecting uptake among adults 18–85 was conducted. The authors found that nationwide coverage was only 4.5% without significant regional differences and about 15–25% in the risk groups such as chronic pulmonary conditions or immunocompromise [40]. Finally, we have also not included the comorbidities of the patients in our analyses despite the fact that comorbidities could be confounding factors in the analysis of mortality. Reassuringly, the serotypes associated with death are similar as compared to our earlier study from 2014 (which included the comorbidities as potential confounders) [9].

## Conclusion

In 2022, the IPD incidence is again equal to or even higher for infants and children after the COVID-19 pandemic. In contrast, numbers have not yet fully rebounded in the 65+ group. However, the COVID-19 pandemic has had almost no effect on the serotype distribution. With the exception of serotype 3, the burden of disease is mainly due to non-PCV13 serotypes. The already existing or planned introduction of higher valent PCVs is expected to contribute to a decreased number of cases and could help to reduce mortality. An ongoing and longer follow-up is needed to confirm these serotype epidemiologic changes, particularly along with the introduction of new vaccines.

## Supplementary Material

supplementary_albrich_just_R2.docx
