# Supplementary material for: Serotype epidemiology and case-fatality risk of invasive pneumococcal disease: a nationwide population study from Switzerland, 2012–2022
Source: Emerg Microbes Infect. 2025 Apr 1;14(1):2488189. doi: 10.1080/22221751.2025.2488189 (PMC12024505; doi:10.1080/22221751.2025.2488189)
Supplement: supplementary_albrich_just_R2.docx [file TEMI_A_2488189_SM6508.docx]

Supplementary tables

| **Supplementary table 1: Distribution of manifestation groups across different age groups, Switzerland 2012-2022** | | | | | | | | | | | | |
| --- | --- | --- | --- | --- | --- | --- | --- | --- | --- | --- | --- | --- |
| No. of cases per 100'000 population  No. of cases (%) | | | | | | | | | | | | |
| Manifestation-group | Year | | | | | | | | | | | IRR (95% CI, p-value)^1^ |
|  | 2012 | 2013 | 2014 | 2015 | 2016 | 2017 | 2018 | 2019 | 2020 | 2021 | 2022 |  |
| Known | 835 (97.0) | 859 (96.4) | 757 (96.3) | 867 (96.3) | 759 (92.4) | 899 (95.3) | 876 (95.2) | 773 (93.6) | 450 (86.9) | 385 (80.0) | 684 (85.5) |  |
| Age 0-16 |  |  |  |  |  |  |  |  |  |  |  |  |
| Pneumonia (without Meningitis) | 1.9 | 2.6 | 1.6 | 1.4 | 1.3 | 1.0 | 2.3 | 1.4 | 0.5 | 1.1 | 1.7 | 0.94 (0.91-0.98, 0.005)^2^ |
|  | 26 (3.1) | 36 (4.2) | 23 (3.0) | 19 (2.2) | 19 (2.5) | 14 (1.6) | 33 (3.8) | 21 (2.7) | 7 (1.6) | 16 (4.2) | 25 (3.7) | 0.97 (0.94-1.01, 0.16)^3^ |
| Meningitis | 0.4 | 0.8 | 0.8 | 0.6 | 0.5 | 0.1 | 0.3 | 0.3 | 0.3 | 0.3 | 1.0 | 0.98 (0.91-1.05, 0.62)^2^ |
|  | 5 (0.6) | 11 (1.3) | 11 (1.5) | 8 (0.9) | 7 (0.9) | 2 (0.2) | 4 (0.5) | 4 (0.5) | 5 (1.1) | 4 (1.0) | 15 (2.2) | 1.01 (0.94-1.08, 0.84)^3^ |
| Bacteremia without focus | 0.5 | 0.4 | 0.1 | 0.6 | 0.1 | 0.2 | 0.4 | 0.3 | 0.3 | 0.2 | 0.4 | 0.98 (0.89-1.06, 0.58)^2^ |
|  | 7 (0.8) | 5 (0.6) | 2 (0.3) | 8 (0.9) | 2 (0.3) | 3 (0.3) | 6 (0.7) | 4 (0.5) | 5 (1.1) | 3 (0.8) | 6 (0.9) | 1.00 (0.92-1.09, 0.98)^3^ |
| Other | 0.5 | 0.5 | 0.4 | 0.5 | 0.3 | 0.7 | 0.6 | 0.2 | 0.3 | 0.7 | 1.1 | 1.06 (0.99-1.13, 0.12)^2^ |
|  | 7 (0.8) | 7 (0.8) | 6 (0.8) | 7 (0.8) | 4 (0.5) | 10 (1.1) | 9 (1.0) | 3 (0.4) | 4 (0.9) | 10 (2.6) | 17 (2.5) | 1.07 (1.01-1.14, 0.03)^3^ |
| Age 17-64 |  |  |  |  |  |  |  |  |  |  |  |  |
| Pneumonia (without Meningitis) | 4.5 | 4.6 | 3.3 | 4.0 | 3.2 | 3.3 | 3.9 | 3.4 | 2.1 | 1.7 | 2.5 | 0.93 (0.92-0.94, <0.001)^2^ |
|  | 238 (28.5) | 247 (28.8) | 175 (23.1) | 217 (25.0) | 173 (22.8) | 182 (20.2) | 217 (24.8) | 186 (24.1) | 116 (25.8) | 96 (24.9) | 143 (20.9) | 0.99 (0.98-1.01, 0.22)^3^ |
| Meningitis | 0.3 | 0.4 | 0.2 | 0.5 | 0.5 | 0.6 | 0.5 | 0.5 | 0.3 | 0.2 | 0.3 | 0.98 (0.94-1.02, 0.39)^2^ |
|  | 17 (2.0) | 22 (2.6) | 11 (1.5) | 25 (2.9) | 28 (3.7) | 31 (3.4) | 25 (2.9) | 26 (3.4) | 16 (3.6) | 13 (3.4) | 16 (2.3) | 1.05 (1.00-1.09, 0.03)^3^ |
| Bacteremia without focus | 0.7 | 0.7 | 0.7 | 0.6 | 0.5 | 0.5 | 0.6 | 0.4 | 0.4 | 0.4 | 0.2 | 0.92 (0.89-0.95, <0.001)^2^ |
|  | 38 (4.6) | 38 (4.4) | 35 (4.6) | 32 (3.7) | 25 (3.3) | 29 (3.2) | 31 (3.5) | 24 (3.1) | 22 (4.9) | 21 (5.5) | 14 (2.0) | 0.98 (0.94-1.01, 0.23)^3^ |
| Other | 0.4 | 0.4 | 0.5 | 0.4 | 0.5 | 0.6 | 0.4 | 0.3 | 0.3 | 0.4 | 0.5 | 0.99 (0.95-1.03, 0.56)^2^ |
|  | 23 (2.8) | 21 (2.4) | 27 (3.6) | 22 (2.5) | 29 (3.8) | 34 (3.8) | 22 (2.5) | 19 (2.5) | 17 (3.8) | 22 (5.7) | 28 (4.1) | 1.05 (1.01-1.10, 0.008)^3^ |
| Age 65+ |  |  |  |  |  |  |  |  |  |  |  |  |
| Pneumonia (without Meningitis) | 26.1 | 25.1 | 22.9 | 26.8 | 23.1 | 29.7 | 26.7 | 23.1 | 11.5 | 9.9 | 19.4 | 0.95 (0.94-0.96, <0.001)^2^ |
|  | 365 (43.7) | 359 (41.8) | 335 (44.3) | 401 (46.3) | 352 (46.4) | 461 (51.3) | 421 (48.1) | 371 (48.0) | 188 (41.8) | 164 (42.6) | 328 (48.0) | 1.00 (0.99-1.02, 0.44)^3^ |
| Meningitis | 1.0 | 1.0 | 1.9 | 1.9 | 1.6 | 2.1 | 1.1 | 1.7 | 0.5 | 0.4 | 1.7 | 0.97 (0.93-1.01, 0.18)^2^ |
|  | 14 (1.7) | 14 (1.6) | 28 (3.7) | 28 (3.2) | 25 (3.3) | 32 (3.6) | 18 (2.1) | 27 (3.5) | 8 (1.8) | 7 (1.8) | 29 (4.2) | 1.04 (0.99-1.08, 0.11)^3^ |
| Bacteremia without focus | 4.6 | 5.1 | 4.6 | 4.9 | 4.1 | 3.5 | 3.6 | 3.0 | 2.1 | 1.0 | 1.8 | 0.89 (0.87-0.91, <0.001)^2^ |
|  | 64 (7.7) | 73 (8.5) | 67 (8.9) | 74 (8.5) | 63 (8.3) | 55 (6.1) | 57 (6.5) | 48 (6.2) | 34 (7.6) | 16 (4.2) | 30 (4.4) | 0.94 (0.91-0.97, <0.001)^3^ |
| Other | 2.2 | 1.8 | 2.5 | 1.7 | 2.1 | 3.0 | 2.0 | 2.5 | 1.7 | 0.8 | 2.0 | 0.97 (0.94-1.00, 0.07)^2^ |
|  | 31 (3.7) | 26 (3.0) | 37 (4.9) | 26 (3.0) | 32 (4.2) | 46 (5.1) | 32 (3.7) | 40 (5.2) | 28 (6.2) | 13 (3.4) | 33 (4.8) | 1.03 (1.00-1.07, 0.07)^3^ |
| ^1^Poisson Regression | | | | | | | | | | | | |
| ^2^N = age group stratified population | | | | | | | | | | | | |
| ^3^N = total number of cases from specific age group with manifestation known | | | | | | | | | | | | |

| **Supplementary table 2: Serotype-groups of *Streptococcus pneumoniae* causing invasive pneumococcal disease (IPD) across different ages, Switzerland 2012-2022** | | | | | | | | | | | | |
| --- | --- | --- | --- | --- | --- | --- | --- | --- | --- | --- | --- | --- |
| No. of cases per 100’000 population,  IPD-cases with serotype known no. (%) | | | | | | | | | | | | |
| Serotype-group | Year | | | | | | | | | | | IRR (95% CI, p-value)^1^ |
|  | 2012 | 2013 | 2014 | 2015 | 2016 | 2017 | 2018 | 2019 | 2020 | 2021 | 2022 |  |
| Total |  |  |  |  |  |  |  |  |  |  |  |  |
| PCV13^2^ | 5.9 | 5.5 | 3.9 | 4.0 | 3.5 | 3.3 | 3.4 | 2.5 | 1.6 | 1.3 | 2.4 | 0.88 (0.87-0.89, <0.001)^3^ |
|  | 478 (61.0) | 448 (54.8) | 321 (45.5) | 331 (39.6) | 293 (37.7) | 280 (32.4) | 287 (35.1) | 211 (28.7) | 136 (29.8) | 111 (26.7) | 210 (33.8) |  |
| PCV15-non-13 | 0.6 | 0.9 | 1.1 | 1.0 | 1.0 | 1.1 | 1.1 | 1.1 | 0.5 | 0.4 | 0.6 | 0.96 (0.94-0.96, <0.001)^3^ |
|  | 51 (6.5) | 76 (9.3) | 90 (12.7) | 84 (10.1) | 84 (10.8) | 95 (11.0) | 95 (11.6) | 95 (12.9) | 43 (9.4) | 37 (8.9) | 52 (8.4) |  |
| PCV20-non-15 | 1.2 | 1.3 | 1.4 | 2.0 | 2.0 | 2.6 | 2.6 | 2.4 | 1.6 | 1.5 | 2.1 | 1.03 (1.02-1.05, <0.001)^3^ |
|  | 99 (12.6) | 109 (13.3) | 114 (16.1) | 165 (19.8) | 170 (21.9) | 223 (25.8) | 223 (27.3) | 205 (27.9) | 136 (29.8) | 130 (31.2) | 184 (29.6) |  |
| NVT^4^ | 1.9 | 2.3 | 2.2 | 3.1 | 2.7 | 3.1 | 2.5 | 2.6 | 1.6 | 1.6 | 2.0 | 0.98 (0.97-0.99, 0.001)^3^ |
|  | 156 (19.9) | 184 (22.5) | 181 (25.6) | 255 (30.5) | 231 (29.7) | 266 (30.8) | 213 (26.0) | 225 (30.6) | 141 (30.9) | 138 (33.2) | 176 (28.3) |  |
| Age 0-4 |  |  |  |  |  |  |  |  |  |  |  |  |
| PCV13 | 4.5 | 3.6 | 2.9 | 2.4 | 2.1 | 1.8 | 4.6 | 1.8 | 0.7 | 1.8 | 2.5 | 0.93 (0.87-0.98, 0.008)^3^ |
|  | 18 (2.3) | 15 (1.8) | 12 (1.7) | 10 (1.2) | 9 (1.2) | 8 (0.9) | 20 (2.4) | 8 (1.1) | 3 (0.7) | 8 (1.9) | 11 (1.8) |  |
| PCV15-non-13 | 0.0 | 1.0 | 0.5 | 0.0 | 0.0 | 0.7 | 0.0 | 0.9 | 0.0 | 0.7 | 0.7 | 1.06 (0.92-1.24, 0.40)^3^ |
|  | 0 (0.0) | 4 (0.5) | 2 (0.3) | 0 (0.0) | 0 (0.0) | 3 (0.3) | 0 (0.0) | 4 (0.5) | 0 (0.0) | 3 (0.7) | 3 (0.5) |  |
| PCV20-non-15 | 1.0 | 1.2 | 1.0 | 1.4 | 0.2 | 0.9 | 1.4 | 0.5 | 1.4 | 2.3 | 1.8 | 1.07 (0.99-1.17, 0.10)^3^ |
|  | 4 (0.5) | 5 (0.6) | 4 (0.6) | 6 (0.7) | 1 (0.1) | 4 (0.5) | 6 (0.7) | 2 (0.3) | 6 (1.3) | 10 (2.4) | 8 (1.3) |  |
| NVT | 2.2 | 1.0 | 1.7 | 1.7 | 2.6 | 1.2 | 1.4 | 1.8 | 1.4 | 1.8 | 2.8 | 1.02 (0.96-1.10, 0.51)^3^ |
|  | 9 (1.1) | 4 (0.5) | 7 (1.0) | 7 (0.8) | 11 (1.4) | 5 (0.6) | 6 (0.7) | 8 (1.1) | 6 (1.3) | 8 (1.9) | 12 (1.9) |  |
| Age 5-16 |  |  |  |  |  |  |  |  |  |  |  |  |
| PCV13 | 1.0 | 2.0 | 1.1 | 1.0 | 0.8 | 0.3 | 1.1 | 0.6 | 0.5 | 0.8 | 1.0 | 0.93 (0.88-0.99, 0.03)^3^ |
|  | 10 (1.3) | 19 (2.3) | 11 (1.6) | 10 (1.2) | 8 (1.0) | 3 (0.3) | 11 (1.3) | 6 (0.8) | 5 (1.1) | 8 (1.9) | 11 (1.8) |  |
| PCV15-non-13 | 0.0 | 0.1 | 0.1 | 0.0 | 0.1 | 0.0 | 0.0 | 0.0 | 0.3 | 0.1 | 0.2 | 1.18 (0.95-1.51, 0.15)^3^ |
|  | 0 (0.0) | 1 (0.1) | 1 (0.1) | 0 (0.0) | 1 (0.1) | 0 (0.0) | 0 (0.0) | 0 (0.0) | 3 (0.7) | 1 (0.2) | 2 (0.3) |  |
| PCV20-non-15 | 0.1 | 0.3 | 0.1 | 0.5 | 0.1 | 0.4 | 0.2 | 0.2 | 0.2 | 0.3 | 0.6 | 1.07 (0.96-1.20, 0.25)^3^ |
|  | 1 (0.1) | 3 (0.4) | 1 (0.1) | 5 (0.6) | 1 (0.1) | 4 (0.5) | 2 (0.2) | 2 (0.3) | 2 (0.4) | 3 (0.7) | 6 (1.0) |  |
| NVT | 0.1 | 0.3 | 0.2 | 0.4 | 0.1 | 0.1 | 0.5 | 0.3 | 0.1 | 0.5 | 0.4 | 1.07 (0.95-1.20, 0.27)^3^ |
|  | 1 (0.1) | 3 (0.4) | 2 (0.3) | 4 (0.5) | 1 (0.1) | 1 (0.1) | 5 (0.6) | 3 (0.4) | 1 (0.2) | 5 (1.2) | 4 (0.6) |  |
| Age 17-64 |  |  |  |  |  |  |  |  |  |  |  |  |
| PCV13 | 3.4 | 3.3 | 2.0 | 2.2 | 1.7 | 1.7 | 1.7 | 1.3 | 0.8 | 0.7 | 1.0 | 0.87 (0.85-0.88, <0.001)^3^ |
|  | 177 (22.6) | 175 (21.4) | 109 (15.4) | 118 (14.1) | 93 (12.0) | 92 (10.6) | 92 (11.2) | 71 (9.6) | 47 (10.3) | 39 (9.4) | 58 (9.3) |  |
| PCV15-non-13 | 0.3 | 0.5 | 0.5 | 0.4 | 0.4 | 0.5 | 0.5 | 0.5 | 0.3 | 0.2 | 0.2 | 0.96 (0.92-1.00, 0.05)^3^ |
|  | 15 (1.9) | 25 (3.1) | 25 (3.5) | 22 (2.6) | 24 (3.1) | 30 (3.5) | 28 (3.4) | 26 (3.5) | 18 (3.9) | 12 (2.9) | 11 (1.8) |  |
| PCV20-non-15 | 0.7 | 0.7 | 0.7 | 1.2 | 1.4 | 1.5 | 1.8 | 1.5 | 1.1 | 0.9 | 1.2 | 1.04 (1.02-1.07, <0.001)^3^ |
|  | 37 (4.7) | 36 (4.4) | 39 (5.5) | 64 (7.7) | 79 (10.2) | 82 (9.5) | 99 (12.1) | 81 (11.0) | 64 (14.0) | 51 (12.3) | 67 (10.8) |  |
| NVT | 1.3 | 1.3 | 1.0 | 1.5 | 1.2 | 1.4 | 1.0 | 1.2 | 0.8 | 0.9 | 0.8 | 0.96 (0.93-0.98, <0.001)^3^ |
|  | 68 (8.7) | 69 (8.4) | 54 (7.6) | 79 (9.5) | 67 (8.6) | 75 (8.7) | 56 (6.8) | 68 (9.2) | 46 (10.1) | 49 (11.8) | 43 (6.9) | ^4^ |
| Age 65+ |  |  |  |  |  |  |  |  |  |  |  |  |
| PCV13 | 19.5 | 16.7 | 12.9 | 12.9 | 12.0 | 11.4 | 10.4 | 7.8 | 5.0 | 3.3 | 7.7 | 0.88 (0.87-0.89, <0.001)^3^ |
|  | 273 (34.8) | 239 (29.3) | 189 (26.8) | 193 (23.1) | 183 (23.5) | 177 (20.5) | 164 (20.0) | 126 (17.1) | 81 (17.8) | 55 (13.2) | 130 (20.9) |  |
| PCV15-non-13 | 2.6 | 3.2 | 4.2 | 4.1 | 3.9 | 4.0 | 4.2 | 4.0 | 1.3 | 1.3 | 2.1 | 0.94 (0.92-0.97, <0.001)^3^ |
|  | 36 (4.6) | 46 (5.6) | 62 (8.8) | 62 (7.4) | 59 (7.6) | 62 (7.2) | 66 (8.1) | 65 (8.8) | 22 (4.8) | 21 (5.0) | 36 (5.8) |  |
| PCV20-non-15 | 4.1 | 4.5 | 4.8 | 6.0 | 5.8 | 8.6 | 7.4 | 7.5 | 3.9 | 4.0 | 6.1 | 1.01 (0.99-1.03, 0.21)^3^ |
|  | 57 (7.3) | 65 (8.0) | 70 (9.9) | 90 (10.8) | 89 (11.4) | 133 (15.4) | 116 (14.2) | 120 (16.3) | 64 (14.0) | 66 (15.9) | 103 (16.6) |  |
| NVT | 5.6 | 7.5 | 8.1 | 11.0 | 10.0 | 11.9 | 9.3 | 9.1 | 5.4 | 4.6 | 6.9 | 0.98 (0.96-0.99, 0.006)^3^ |
|  | 78 (9.9) | 108 (13.2) | 118 (16.7) | 165 (19.8) | 152 (19.5) | 185 (21.4) | 146 (17.8) | 146 (19.8) | 88 (19.3) | 76 (18.3) | 117 (18.8) |  |
| ^1^Poisson Regression | | | | | | | | | | | | |
| ^2^Pneumococcal conjugate vaccine | | | | | | | | | | | | |
| ^3^N = total population or age group stratified population | | | | | | | | | | | | |
| 4Non-vaccine serotypes, i.e. serotypes not covered by PCV20 | | | | | | | | | | | | |

| **Supplementary table 3: Serotype-groups of Streptococcus pneumoniae causing invasive pneumococcal disease (IPD) across different ages, Switzerland 2019-2022** | | | | | |
| --- | --- | --- | --- | --- | --- |
| IPD-cases with serotype known, no. (%) | | | | | |
| Serotype-group | Year | | | | X^2^ (p-value)^1^ |
|  | 2019 | 2020 | 2021 | 2022 |  |
| Total |  |  |  |  |  |
| PCV13^2^ | 211 (28.7) | 136 (29.8) | 111 (26.7) | 210 (33.8) | 3.85 (0.05) |
| PCV15 | 306 (41.6) | 179 (39.3) | 148 (35.6) | 262 (42.1) | 0.02 (0.88) |
| PCV20 | 505 (68.6) | 308 (67.5) | 276 (66.3) | 443 (71.2) | 0.97 (0.33) |
| V116^3^ | 608 (82.6) | 363 (79.6) | 346 (83.2) | 508 (81.7) | 0.14 (0.71) |
| Age 0-16 |  |  |  |  |  |
| PCV13 | 14 (42.4) | 8 (30.8) | 16 (34.8) | 22 (38.6) | 0.02 (0.89) |
| PCV15 | 18 (54.5) | 11 (42.3) | 20 (43.5) | 27 (47.4) | 0.19 (0.66) |
| PCV20 | 22 (66.7) | 18 (69.2) | 32 (69.6) | 39 (68.4) | 0.00 (1.0) |
| V116 | 28 (84.8) | 21 (80.8) | 33 (71.7) | 41 (71.9) | 1.29 (0.26) |
| Age 17-64 |  |  |  |  |  |
| PCV13 | 71 (28.9) | 47 (26.9) | 39 (25.8) | 58 (32.4) | 0.46 (0.5) |
| PCV15 | 97 (39.4) | 65 (37.1) | 51 (33.8) | 69 (38.5) | 0.01 (0.93) |
| PCV20 | 177 (72) | 126 (72) | 102 (67.5) | 136 (76) | 0.67 (0.41) |
| V116 | 204 (82.9) | 145 (82.9) | 126 (83.4) | 151 (84.4) | 0.07 (0.79) |
| Age 65+ |  |  |  |  |  |
| PCV13 | 126 (27.6) | 81 (31.8) | 55 (25.2) | 130 (33.7) | 3.41 (0.06) |
| PCV15 | 191 (41.8) | 103 (40.4) | 76 (34.9) | 166 (43) | 0.08 (0.78) |
| PCV20 | 306 (67) | 164 (64.3) | 141 (64.7) | 268 (69.4) | 0.48 (0.49) |
| V116 | 376 (82.3) | 197 (77.3) | 186 (85.3) | 316 (81.9) | 0.00 (0.95) |
| ^1^2x2 Pearson’s Chi-squared Test with Yates continuity correction, 2019 vs 2022 | | | | | |
| ^2^Pneumococcal conjugate vaccine | | | | | |
| ^3^Serotyp 20 instead of 20A | | | | | |

| **Supplementary table 4: Serotypes of *Streptococcus pneumoniae* causing invasive pneumococcal disease (IPD) in 17-64 age-group, Switzerland 2012-2022** | | | | | | | | | | | | |
| --- | --- | --- | --- | --- | --- | --- | --- | --- | --- | --- | --- | --- |
| No. of cases per 100'000 population,  IPD-cases with serotype known no. (%) | | | | | | | | | | | | |
| Serotype | Year | | | | | | | | | | | IRR (95% CI, p-value)^1^ |
|  | 2012 | 2013 | 2014 | 2015 | 2016 | 2017 | 2018 | 2019 | 2020 | 2021 | 2022 |  |
| Known | 297 (91.4) | 305 (89.4) | 227 (87.6) | 283 (92.2) | 263 (96.0) | 279 (94.9) | 275 (88.1) | 246 (91.8) | 175 (87.9) | 151 (85.3) | 179 (77.2) |  |
| PCV13^2^ |  |  |  |  |  |  |  |  |  |  |  |  |
| 1 | 0.34 | 0.26 | 0.15 | 0.06 | 0.02 | 0.05 | 0.00 | 0.00 | 0.00 | 0.00 | 0.00 | 0.55 (0.45-0.65, <0.001)^3^ |
|  | 18 (6.1) | 14 (4.6) | 8 (3.5) | 3 (1.1) | 1 (0.4) | 3 (1.1) | 0 (0.0) | 0 (0.0) | 0 (0.0) | 0 (0.0) | 0 (0.0) | 0.58 (0.48-0.68, <0.001)^4^ |
| 3 | 0.80 | 1.03 | 0.76 | 1.01 | 0.69 | 0.73 | 0.92 | 0.60 | 0.48 | 0.45 | 0.59 | 0.94 (0.91-0.97, <0.001)^3^ |
|  | 42 (14.1) | 55 (18.0) | 41 (18.1) | 55 (19.4) | 38 (14.4) | 40 (14.3) | 51 (18.5) | 33 (13.4) | 27 (15.4) | 25 (16.6) | 33 (18.4) | 1.00, (0.97-1.03, 0.947)^4^ |
| 4 | 0.15 | 0.23 | 0.17 | 0.15 | 0.20 | 0.22 | 0.11 | 0.09 | 0.05 | 0.02 | 0.12 | 0.90 (0.84-0.97, 0.004)^3^ |
|  | 8 (2.7) | 12 (3.9) | 9 (4.0) | 8 (2.8) | 11 (4.2) | 12 (4.3) | 6 (2.2) | 5 (2.0) | 3 (1.7) | 1 (0.7) | 7 (3.9) | 0.95 (0.89-1.03, 0.211)^4^ |
| 5 | 0.02 | 0.08 | 0.04 | 0.00 | 0.00 | 0.00 | 0.00 | 0.00 | 0.00 | 0.00 | 0.00 | 0.53 (0.29-0.78, 0.010)^3^ |
|  | 1 (0.3) | 4 (1.3) | 2 (0.9) | 0 (0.0) | 0 (0.0) | 0 (0.0) | 0 (0.0) | 0 (0.0) | 0 (0.0) | 0 (0.0) | 0 (0.0) | 0.56 (0.30-0.82, 0.016)^4^ |
| 6A | 0.09 | 0.08 | 0.09 | 0.07 | 0.04 | 0.00 | 0.02 | 0.00 | 0.02 | 0.00 | 0.00 | 0.70 (0.58-0.83, <0.001)^3^ |
|  | 5 (1.7) | 4 (1.3) | 5 (2.2) | 4 (1.4) | 2 (0.8) | 0 (0.0) | 1 (0.4) | 0 (0.0) | 1 (0.6) | 0 (0.0) | 0 (0.0) | 0.74 (0.61-0.87, <0.001)^4^ |
| 6B | 0.04 | 0.04 | 0.02 | 0.00 | 0.02 | 0.05 | 0.00 | 0.02 | 0.02 | 0.02 | 0.00 | 0.89 (0.73-1.07, 0.215)^3^ |
|  | 2 (0.7) | 2 (0.7) | 1 (0.4) | 0 (0.0) | 1 (0.4) | 3 (1.1) | 0 (0.0) | 1 (0.4) | 1 (0.6) | 1 (0.7) | 0 (0.0) | 0.94 (0.77-1.13, 0.530)^4^ |
| 7F | 0.84 | 0.58 | 0.20 | 0.24 | 0.24 | 0.11 | 0.07 | 0.05 | 0.02 | 0.00 | 0.02 | 0.66 (0.61-0.71, <0.001)^3^ |
|  | 44 (14.8) | 31 (10.2) | 11 (4.8) | 13 (4.6) | 13 (4.9) | 6 (2.2) | 4 (1.5) | 3 (1.2) | 1 (0.6) | 0 (0.0) | 1 (0.6) | 0.69 (0.63-0.75, <0.001)^4^ |
| 9V | 0.13 | 0.09 | 0.04 | 0.04 | 0.02 | 0.05 | 0.04 | 0.07 | 0.00 | 0.00 | 0.02 | 0.81 (0.70-0.92, 0.002)^3^ |
|  | 7 (2.4) | 5 (1.6) | 2 (0.9) | 2 (0.7) | 1 (0.4) | 3 (1.1) | 2 (0.7) | 4 (1.6) | 0 (0.0) | 0 (0.0) | 1 (0.6) | 0.85 (0.74-0.97, 0.023)^4^ |
| 14 | 0.15 | 0.21 | 0.13 | 0.04 | 0.05 | 0.05 | 0.09 | 0.13 | 0.07 | 0.02 | 0.02 | 0.86 (0.78-0.94, 0.001)^3^ |
|  | 8 (2.7) | 11 (3.6) | 7 (3.1) | 2 (0.7) | 3 (1.1) | 3 (1.1) | 5 (1.8) | 7 (2.8) | 4 (2.3) | 1 (0.7) | 1 (0.6) | 0.91 (0.82-1.00, 0.044)^4^ |
| 18C | 0.09 | 0.04 | 0.07 | 0.07 | 0.02 | 0.00 | 0.02 | 0.07 | 0.02 | 0.02 | 0.02 | 0.86 (0.75-0.98, 0.029)^3^ |
|  | 5 (1.7) | 2 (0.7) | 4 (1.8) | 4 (1.4) | 1 (0.4) | 0 (0.0) | 1 (0.4) | 4 (1.6) | 1 (0.6) | 1 (0.7) | 1 (0.6) | 0.91 (0.79-1.04, 0.177)^4^ |
| 19A | 0.44 | 0.47 | 0.30 | 0.41 | 0.29 | 0.27 | 0.18 | 0.16 | 0.09 | 0.09 | 0.16 | 0.86 (0.81-0.91, <0.001)^3^ |
|  | 23 (7.7) | 25 (8.2) | 16 (7.0) | 22 (7.8) | 16 (6.1) | 15 (5.4) | 10 (3.6) | 9 (3.7) | 5 (2.9) | 5 (3.3) | 9 (5.0) | 0.91 (0.86-0.96, <0.001)^4^ |
| 19F | 0.06 | 0.06 | 0.04 | 0.04 | 0.09 | 0.11 | 0.18 | 0.09 | 0.05 | 0.09 | 0.09 | 1.06 (0.97, 1.16, 0.191)^3^ |
|  | 3 (1.0) | 3 (1.0) | 2 (0.9) | 2 (0.7) | 5 (1.9) | 6 (2.2) | 10 (3.6) | 5 (2.0) | 3 (1.7) | 5 (3.3) | 5 (2.8) | 1.14 (1.04-1.25, 0.007)^4^ |
| 23F | 0.21 | 0.13 | 0.02 | 0.06 | 0.02 | 0.02 | 0.04 | 0.00 | 0.02 | 0.00 | 0.00 | 0.64 (0.52-0.76, <0.001)^3^ |
|  | 11 (3.7) | 7 (2.3) | 1 (0.4) | 3 (1.1) | 1 (0.4) | 1 (0.4) | 2 (0.7) | 0 (0.0) | 1 (0.6) | 0 (0.0) | 0 (0.0) | 0.67 (0.55-0.80, <0.001)^4^ |
| PCV15-non-PCV13 |  |  |  |  |  |  |  |  |  |  |  |  |
| 22F | 0.27 | 0.45 | 0.39 | 0.35 | 0.40 | 0.45 | 0.49 | 0.42 | 0.25 | 0.16 | 0.16 | 0.95 (0.91-0.99, 0.020)^3^ |
|  | 14 (4.7) | 24 (7.9) | 21 (9.3) | 19 (6.7) | 22 (8.4) | 25 (9.0) | 27 (9.8) | 23 (9.3) | 14 (8.0) | 9 (6.0) | 9 (5.0) | 1.01 (0.96-1.05, 0.697)^4^ |
| 33F | 0.02 | 0.02 | 0.07 | 0.06 | 0.04 | 0.09 | 0.02 | 0.05 | 0.07 | 0.05 | 0.04 | 1.04 (0.92-1.17, 0.548)^3^ |
|  | 1 (0.3) | 1 (0.3) | 4 (1.8) | 3 (1.1) | 2 (0.8) | 5 (1.8) | 1 (0.4) | 3 (1.2) | 4 (2.3) | 3 (2.0) | 2 (1.1) | 1.11 (0.98-1.25, 0.097)^4^ |
| PCV20-non-PCV15 |  |  |  |  |  |  |  |  |  |  |  |  |
| 8 | 0.42 | 0.43 | 0.32 | 0.72 | 0.91 | 0.91 | 1.01 | 0.88 | 0.79 | 0.7 | 1.00 | 1.07 (1.04-1.11, <0.001)^3^ |
|  | 22 (7.4) | 23 (7.5) | 17 (7.5) | 39 (13.8) | 50 (19.0) | 50 (17.9) | 56 (20.4) | 49 (19.9) | 44 (25.1) | 39 (25.8) | 56 (31.3) | 1.15 (1.12-1.19, <0.001)^4^ |
| 10A | 0.08 | 0.06 | 0.07 | 0.07 | 0.13 | 0.18 | 0.14 | 0.11 | 0.13 | 0.07 | 0.04 | 1.01 (0.93-1.09, 0.857)^3^ |
|  | 4 (1.3) | 3 (1.0) | 4 (1.8) | 4 (1.4) | 7 (2.7) | 10 (3.6) | 8 (2.9) | 6 (2.4) | 7 (4.0) | 4 (2.6) | 2 (1.1) | 1.07 (0.99-1.17, 0.093)^4^ |
| 11A | 0.08 | 0.08 | 0.07 | 0.09 | 0.15 | 0.09 | 0.09 | 0.05 | 0.04 | 0.07 | 0.05 | 0.96 (0.87-1.05, 0.335)^3^ |
|  | 4 (1.3) | 4 (1.3) | 4 (1.8) | 5 (1.8) | 8 (3.0) | 5 (1.8) | 5 (1.8) | 3 (1.2) | 2 (1.1) | 4 (2.6) | 3 (1.7) | 1.02 (0.92-1.12, 0.738)^4^ |
| 12F | 0.06 | 0.09 | 0.17 | 0.22 | 0.11 | 0.20 | 0.43 | 0.34 | 0.07 | 0.04 | 0.07 | 1.00 (0.94-1.07, 0.901)^3^ |
|  | 3 (1.0) | 5 (1.6) | 9 (4.0) | 12 (4.2) | 6 (2.3) | 11 (3.9) | 24 (8.7) | 19 (7.7) | 4 (2.3) | 2 (1.3) | 4 (2.2) | 1.07 (1.00-1.14, 0.039)^4^ |
| 15B/C^5^ | 0.08 | 0.02 | 0.09 | 0.07 | 0.15 | 0.11 | 0.11 | 0.07 | 0.13 | 0.04 | 0.04 | 0.99 (0.91-1.08, 0.820)^3^ |
|  | 4 (1.3) | 1 (0.3) | 5 (2.2) | 4 (1.4) | 8 (3.0) | 6 (2.2) | 6 (2.2) | 4 (1.6) | 7 (4.0) | 2 (1.3) | 2 (1.1) | 1.05 (0.96-1.16, 0.259)^4^ |
| NVT^6^ |  |  |  |  |  |  |  |  |  |  |  |  |
| 10B | 0.04 | 0.00 | 0.00 | 0.02 | 0.04 | 0.04 | 0.04 | 0.00 | 0.02 | 0.02 | 0.02 | 0.99 (0.83-1.19, 0.946)^3^ |
|  | 2 (0.7) | 0 (0.0) | 0 (0.0) | 1 (0.4) | 2 (0.8) | 2 (0.7) | 2 (0.7) | 0 (0.0) | 1 (0.6) | 1 (0.7) | 1 (0.6) | 1.06 (0.88-1.28, 0.546)^4^ |
| 15A | 0.04 | 0.00 | 0.06 | 0.11 | 0.09 | 0.11 | 0.11 | 0.13 | 0.13 | 0.04 | 0.07 | 1.07 (0.97-1.17, 0.171)^3^ |
|  | 2 (0.7) | 0 (0.0) | 3 (1.3) | 6 (2.1) | 5 (1.9) | 6 (2.2) | 6 (2.2) | 7 (2.8) | 7 (4.0) | 2 (1.3) | 4 (2.2) | 1.14 (1.04-1.25, 0.006)^4^ |
| 16/F^5^ | 0.02 | 0.00 | 0.06 | 0.04 | 0.02 | 0.02 | 0.02 | 0.09 | 0.04 | 0.02 | 0.05 | 1.08 (0.94-1.26, 0.267)^3^ |
|  | 1 (0.3) | 0 (0.0) | 3 (1.3) | 2 (0.7) | 1 (0.4) | 1 (0.4) | 1 (0.4) | 5 (2.0) | 2 (1.1) | 1 (0.7) | 3 (1.7) | 1.16 (1.00-1.35, 0.046)^4^ |
| 17F | 0.02 | 0.04 | 0.00 | 0.07 | 0.00 | 0.02 | 0.02 | 0.07 | 0.00 | 0.04 | 0.00 | 0.97 (0.82-1.14, 0.687)^3^ |
|  | 1 (0.3) | 2 (0.7) | 0 (0.0) | 4 (1.4) | 0 (0.0) | 1 (0.4) | 1 (0.4) | 4 (1.6) | 0 (0.0) | 2 (1.3) | 0 (0.0) | 1.03 (0.87-1.22, 0.736)^4^ |
| 20 | 0.04 | 0.06 | 0.06 | 0.13 | 0.04 | 0.13 | 0.05 | 0.09 | 0.02 | 0.00 | 0.05 | 0.95 (0.86-1.05, 0.337)^3^ |
|  | 2 (0.7) | 3 (1.0) | 3 (1.3) | 7 (2.5) | 2 (0.8) | 7 (2.5) | 3 (1.1) | 5 (2.0) | 1 (0.6) | 0 (0.0) | 3 (1.7) | 1.01 (0.91-1.12, 0.864)^4^ |
| 23A | 0.08 | 0.11 | 0.07 | 0.06 | 0.05 | 0.05 | 0.05 | 0.11 | 0.09 | 0.07 | 0.07 | 1.00 (0.91-1.09, 0.933)^3^ |
|  | 4 (1.3) | 6 (2.0) | 4 (1.8) | 3 (1.1) | 3 (1.1) | 3 (1.1) | 3 (1.1) | 6 (2.4) | 5 (2.9) | 4 (2.6) | 4 (2.2) | 1.06 (0.96-1.17, 0.223)^4^ |
| 23B | 0.06 | 0.13 | 0.15 | 0.24 | 0.15 | 0.27 | 0.22 | 0.14 | 0.04 | 0.18 | 0.02 | 0.97 (0.91-1.04, 0.353)^3^ |
|  | 3 (1.0) | 7 (2.3) | 8 (3.5) | 13 (4.6) | 8 (3.0) | 15 (5.4) | 12 (4.4) | 8 (3.3) | 2 (1.1) | 10 (6.6) | 1 (0.6) | 1.03 (0.96-1.10, 0.393)^4^ |
| 24/F^5^ | 0.04 | 0.09 | 0.09 | 0.18 | 0.16 | 0.07 | 0.04 | 0.04 | 0.07 | 0.04 | 0.07 | 0.94 (0.86-1.03, 0.162)^3^ |
|  | 2 (0.7) | 5 (1.6) | 5 (2.2) | 10 (3.5) | 9 (3.4) | 4 (1.4) | 2 (0.7) | 2 (0.8) | 4 (2.3) | 2 (1.3) | 4 (2.2) | 1.00 (0.91-1.09, 0.926)^4^ |
| 31 | 0.06 | 0.00 | 0.02 | 0.02 | 0.04 | 0.07 | 0.02 | 0.04 | 0.02 | 0.04 | 0.05 | 1.04 (0.90-1.20, 0.583)^3^ |
|  | 3 (1) | 0 (0.0) | 1 (0.4) | 1 (0.4) | 2 (0.8) | 4 (1.4) | 1 (0.4) | 2 (0.8) | 1 (0.6) | 2 (1.3) | 3 (1.7) | 1.11 (0.96-1.29, 0.152)^4^ |
| 35B | 0.06 | 0.02 | 0.02 | 0.07 | 0.02 | 0.09 | 0.04 | 0.02 | 0.05 | 0.07 | 0.02 | 1.01 (0.89-1.14, 0.931)^3^ |
|  | 3 (1.0) | 1 (0.3) | 1 (0.4) | 4 (1.4) | 1 (0.4) | 5 (1.8) | 2 (0.7) | 1 (0.4) | 3 (1.7) | 4 (2.6) | 1 (0.6) | 1.07 (0.94-1.22, 0.279)^4^ |
| 35F | 0.11 | 0.06 | 0.13 | 0.09 | 0.04 | 0.05 | 0.05 | 0.07 | 0.05 | 0.07 | 0.05 | 0.94 (0.85-1.03, 0.200)^3^ |
|  | 6 (2.0) | 3 (1.0) | 7 (3.1) | 5 (1.8) | 2 (0.8) | 3 (1.1) | 3 (1.1) | 4 (1.6) | 3 (1.7) | 4 (2.6) | 3 (1.7) | 1.00 (0.90-1.10, 0.954)^4^ |
| 38 | 0.02 | 0.09 | 0.09 | 0.04 | 0.02 | 0.02 | 0.05 | 0.02 | 0.04 | 0.00 | 0.02 | 0.87 (0.75-0.99, 0.045)^3^ |
|  | 1 (0.3) | 5 (1.6) | 5 (2.2) | 2 (0.7) | 1 (0.4) | 1 (0.4) | 3 (1.1) | 1 (0.4) | 2 (1.1) | 0 (0.0) | 1 (0.6) | 0.92 (0.79-1.05, 0.227)^4^ |
| 6C | 0.13 | 0.13 | 0.04 | 0.11 | 0.04 | 0.09 | 0.07 | 0.02 | 0.04 | 0.04 | 0.02 | 0.85 (0.76-0.94, 0.003)^3^ |
|  | 7 (2.4) | 7 (2.3) | 2 (0.9) | 6 (2.1) | 2 (0.8) | 5 (1.8) | 4 (1.5) | 1 (0.4) | 2 (1.1) | 2 (1.3) | 1 (0.6) | 0.90 (0.80-1.00, 0.052)^4^ |
| 7 | 0.00 | 0.04 | 0.00 | 0.00 | 0.00 | 0.00 | 0.00 | 0.02 | 0.02 | 0.05 | 0.04 | 1.26 (1.00-1.64, 0.063)^3^ |
|  | 0 (0.0) | 2 (0.7) | 0 (0.0) | 0 (0.0) | 0 (0.0) | 0 (0.0) | 0 (0.0) | 1 (0.4) | 1 (0.6) | 3 (2.0) | 2 (1.1) | 1.36 (1.08-1.79, 0.015)^4^ |
| 9N | 0.32 | 0.17 | 0.17 | 0.20 | 0.42 | 0.22 | 0.13 | 0.27 | 0.14 | 0.14 | 0.16 | 0.95 (0.90-1.01, 0.079)^3^ |
|  | 17 (5.7) | 9 (3.0) | 9 (4.0) | 11 (3.9) | 23 (8.7) | 12 (4.3) | 7 (2.5) | 15 (6.1) | 8 (4.6) | 8 (5.3) | 9 (5.0) | 1.01 (0.95-1.07, 0.705)^4^ |
| Other^7^ | 0.27 | 0.36 | 0.06 | 0.07 | 0.11 | 0.11 | 0.11 | 0.11 | 0.07 | 0.07 | 0.05 | 0.85 (0.79-0.92, <0.001)^3^ |
|  | 14 (4.7) | 19 (6.2) | 3 (1.3) | 4 (1.4) | 6 (2.3) | 6 (2.2) | 6 (2.2) | 6 (2.4) | 4 (2.3) | 4 (2.6) | 3 (1.7) | 0.90 (0.83-0.97, 0.010)^4^ |
| ^1^Poisson Regression | | | | | | | | | | | | |
| ^2^Pneumococcal conjugate vaccine | | | | | | | | | | | | |
| ^3^N = 17-64 age-group population | | | | | | | | | | | | |
| ^4^N = total number of cases from 17-64 age-group with serotype known | | | | | | | | | | | | |
| ^5^15B/C = 15B, 15C. 16/F = 16, 16F. 24/F = 24, 24F. | | | | | | | | | | | | |
| ^6^Non-vaccine serotypes, i.e. not covered by PCV20 | | | | | | | | | | | | |

| **Supplementary table 5: Serotypes of Streptococcus pneumoniae causing invasive pneumococcal disease (IPD) in 65+ age-group, Switzerland 2012-2022** | | | | | | | | | | | | |
| --- | --- | --- | --- | --- | --- | --- | --- | --- | --- | --- | --- | --- |
| No. of cases per 100’000 population,  IPD-cases with serotype known, no. (%) | | | | | | | | | | | | |
| Serotype | Year | | | | | | | | | | | IRR (95% CI, p-value)^1^ |
|  | 2012 | 2013 | 2014 | 2015 | 2016 | 2017 | 2018 | 2019 | 2020 | 2021 | 2022 |  |
| Known | 444 (91) | 458 (93.5) | 439 (91.1) | 510 (93.4) | 483 (94) | 557 (90.1) | 492 (89.5) | 457 (87.5) | 255 (87.6) | 218 (85.5) | 386 (78) |  |
| PCV13^2^ |  |  |  |  |  |  |  |  |  |  |  |  |
| 1 | 0.57 | 0.28 | 0.48 | 0.13 | 0.20 | 0.00 | 0.00 | 0.06 | 0.00 | 0.00 | 0.00 | 0.63 (0.51-0.75, <0.001)^3^ |
|  | 8 (1.8) | 4 (0.9) | 7 (1.6) | 2 (0.4) | 3 (0.6) | 0 (0.0) | 0 (0.0) | 1 (0.2) | 0 (0.0) | 0 (0.0) | 0 (0.0) | 0.64 (0.51-0.77, <0.001)^4^ |
| 3 | 5.15 | 4.75 | 4.16 | 4.88 | 6.76 | 5.87 | 6.34 | 4.67 | 2.52 | 2.23 | 5.38 | 0.97 (0.95-0.99, 0.008)^3^ |
|  | 72 (16.2) | 68 (14.8) | 61 (13.9) | 73 (14.3) | 103 (21.3) | 91 (16.3) | 100 (20.3) | 75 (16.4) | 41 (16.1) | 37 (17.0) | 91 (23.6) | 1.03 (1.01-1.06, 0.005)^4^ |
| 4 | 1.22 | 0.77 | 0.61 | 0.67 | 0.46 | 0.45 | 0.32 | 0.12 | 0.18 | 0.30 | 0.24 | 0.83 (0.77-0.90, <0.001)^3^ |
|  | 17 (3.8) | 11 (2.4) | 9 (2.1) | 10 (2.0) | 7 (1.4) | 7 (1.3) | 5 (1.0) | 2 (0.4) | 3 (1.2) | 5 (2.3) | 4 (1.0) | 0.87 (0.80-0.94, <0.001)^4^ |
| 5 | 0.07 | 0.00 | 0.07 | 0.07 | 0.00 | 0.00 | 0.00 | 0.00 | 0.00 | 0.00 | 0.00 | 0.62 (0.29-0.98, 0.10)^3^ |
|  | 1 (0.2) | 0 (0.0) | 1 (0.2) | 1 (0.2) | 0 (0.0) | 0 (0.0) | 0 (0.0) | 0 (0.0) | 0 (0.0) | 0 (0.0) | 0 (0.0) | 0.63 (0.29-1.02, 0.13)^4^ |
| 6A | 1.07 | 0.70 | 0.75 | 0.47 | 0.26 | 0.32 | 0.19 | 0.06 | 0.12 | 0.00 | 0.06 | 0.73 (0.65-0.80, <0.001)^3^ |
|  | 15 (3.4) | 10 (2.2) | 11 (2.5) | 7 (1.4) | 4 (0.8) | 5 (0.9) | 3 (0.6) | 1 (0.2) | 2 (0.8) | 0 (0.0) | 1 (0.3) | 0.75 (0.67-0.83, <0.001)^4^ |
| 6B | 0.57 | 0.21 | 0.41 | 0.13 | 0.26 | 0.32 | 0.13 | 0.12 | 0.12 | 0.00 | 0.12 | 0.83 (0.74-0.93, 0.001)^3^ |
|  | 8 (1.8) | 3 (0.7) | 6 (1.4) | 2 (0.4) | 4 (0.8) | 5 (0.9) | 2 (0.4) | 2 (0.4) | 2 (0.8) | 0 (0.0) | 2 (0.5) | 0.87 (0.77-0.98, 0.02)^4^ |
| 7F | 2.79 | 2.79 | 1.64 | 1.27 | 0.53 | 0.58 | 0.13 | 0.12 | 0.12 | 0.06 | 0.12 | 0.67 (0.62-0.72, <0.001)^3^ |
|  | 39 (8.8) | 40 (8.7) | 24 (5.5) | 19 (3.7) | 8 (1.7) | 9 (1.6) | 2 (0.4) | 2 (0.4) | 2 (0.8) | 1 (0.5) | 2 (0.5) | 0.68 (0.63-0.74, <0.001)^4^ |
| 9V | 0.29 | 0.84 | 0.14 | 0.27 | 0.33 | 0.26 | 0.25 | 0.31 | 0.00 | 0.00 | 0.06 | 0.82 (0.74-0.91, <0.001)^3^ |
|  | 4 (0.9) | 12 (2.6) | 2 (0.5) | 4 (0.8) | 5 (1.0) | 4 (0.7) | 4 (0.8) | 5 (1.1) | 0 (0.0) | 0 (0.0) | 1 (0.3) | 0.86 (0.77-0.96, 0.008)^4^ |
| 14 | 1.36 | 1.81 | 1.43 | 1.74 | 0.66 | 0.77 | 0.76 | 0.62 | 0.49 | 0.06 | 0.06 | 0.81 (0.77-0.86, <0.001)^3^ |
|  | 19 (4.3) | 26 (5.7) | 21 (4.8) | 26 (5.1) | 10 (2.1) | 12 (2.2) | 12 (2.4) | 10 (2.2) | 8 (3.1) | 1 (0.5) | 1 (0.3) | 0.85 (0.80-0.90, <0.001)^4^ |
| 18C | 0.43 | 0.28 | 0.34 | 0.00 | 0.20 | 0.06 | 0.19 | 0.25 | 0.12 | 0.06 | 0.00 | 0.84 (0.74-0.95, 0.006)^3^ |
|  | 6 (1.4) | 4 (0.9) | 5 (1.1) | 0 (0.0) | 3 (0.6) | 1 (0.2) | 3 (0.6) | 4 (0.9) | 2 (0.8) | 1 (0.5) | 0 (0.0) | 0.88 (0.77-1.00, 0.06)^4^ |
| 19A | 4.43 | 2.51 | 1.57 | 2.27 | 1.44 | 2.00 | 1.58 | 0.62 | 0.92 | 0.24 | 0.71 | 0.83 (0.79-0.86, <0.001)^3^ |
|  | 62 (14.0) | 36 (7.9) | 23 (5.2) | 34 (6.7) | 22 (4.6) | 31 (5.6) | 25 (5.1) | 10 (2.2) | 15 (5.9) | 4 (1.8) | 12 (3.1) | 0.86 (0.83-0.90, <0.001)^4^ |
| 19F | 0.50 | 1.33 | 0.96 | 0.67 | 0.79 | 0.65 | 0.44 | 0.75 | 0.25 | 0.30 | 0.83 | 0.94 (0.88-0.99, 0.03)^3^ |
|  | 7 (1.6) | 19 (4.1) | 14 (3.2) | 10 (2.0) | 12 (2.5) | 10 (1.8) | 7 (1.4) | 12 (2.6) | 4 (1.6) | 5 (2.3) | 14 (3.6) | 0.99 (0.93-1.06, 0.85)^4^ |
| 23F | 1.07 | 0.42 | 0.34 | 0.33 | 0.13 | 0.13 | 0.06 | 0.12 | 0.12 | 0.06 | 0.12 | 0.75 (0.67-0.84, <0.001)^3^ |
|  | 15 (3.4) | 6 (1.3) | 5 (1.1) | 5 (1.1) | 2 (0.4) | 2 (0.4) | 1 (0.2) | 2 (0.4) | 2 (0.8) | 1 (0.5) | 2 (0.5) | 0.78 (0.68-0.87, <0.001)^4^ |
| PCV15-non-PCV13 |  |  |  |  |  |  |  |  |  |  |  |  |
| 22F | 2.50 | 3.00 | 3.75 | 3.41 | 3.35 | 3.29 | 3.61 | 3.55 | 1.17 | 1.08 | 1.89 | 0.94 (0.91-0.97, <0.001)^3^ |
|  | 35 (7.9) | 43 (9.4) | 55 (12.5) | 51 (10.0) | 51 (10.6) | 51 (9.2) | 57 (11.6) | 57 (12.5) | 19 (7.5) | 18 (8.3) | 32 (8.3) | 1.00 (0.97-1.03, 0.81)^4^ |
| 33F | 0.07 | 0.21 | 0.48 | 0.74 | 0.53 | 0.71 | 0.57 | 0.50 | 0.18 | 0.18 | 0.24 | 0.98 (0.91-1.06, 0.58)^3^ |
|  | 1 (0.2) | 3 (0.7) | 7 (1.6) | 11 (2.2) | 8 (1.7) | 11 (2.0) | 9 (1.8) | 8 (1.8) | 3 (1.2) | 3 (1.4) | 4 (1.0) | 1.04 (0.96-1.13, 0.30)^4^ |
| PCV20-non-PCV15 |  |  |  |  |  |  |  |  |  |  |  |  |
| 8 | 1.64 | 2.09 | 1.98 | 2.68 | 3.28 | 4.90 | 4.69 | 3.92 | 2.02 | 2.83 | 4.20 | 1.06 (1.03-1.09, <0.001)^3^ |
|  | 23 (5.2) | 30 (6.6) | 29 (6.6) | 40 (7.8) | 50 (10.4) | 76 (13.6) | 74 (15.0) | 63 (13.8) | 33 (12.9) | 47 (21.6) | 71 (18.4) | 1.13 (1.10-1.17, <0.001)^4^ |
| 10A | 0.57 | 0.70 | 0.96 | 0.94 | 0.72 | 0.90 | 0.51 | 0.81 | 0.43 | 0.24 | 0.65 | 0.95 (0.90-1.01, 0.10)^3^ |
|  | 8 (1.8) | 10 (2.2) | 14 (3.2) | 14 (2.7) | 11 (2.3) | 14 (2.5) | 8 (1.6) | 13 (2.8) | 7 (2.7) | 4 (1.8) | 11 (2.8) | 1.01 (0.95-1.07, 0.73)^4^ |
| 11A | 0.57 | 0.84 | 0.75 | 1.0 | 0.72 | 1.03 | 0.7 | 0.75 | 0.61 | 0.48 | 0.41 | 0.96 (0.90-1.01, 0.13)^3^ |
|  | 8 (1.8) | 12 (2.6) | 11 (2.5) | 15 (2.9) | 11 (2.3) | 16 (2.9) | 11 (2.2) | 12 (2.6) | 10 (3.9) | 8 (3.7) | 7 (1.8) | 1.02 (0.96-1.08, 0.57)^4^ |
| 12F | 0.64 | 0.56 | 0.41 | 0.74 | 0.59 | 0.84 | 0.76 | 1.37 | 0.25 | 0.18 | 0.30 | 0.96 (0.90-1.02, 0.21)^3^ |
|  | 9 (2.0) | 8 (1.7) | 6 (1.4) | 11 (2.2) | 9 (1.9) | 13 (2.3) | 12 (2.4) | 22 (4.8) | 4 (1.6) | 3 (1.4) | 5 (1.3) | 1.02 (0.96-1.09, 0.50)^4^ |
| 15B/C^5^ | 0.64 | 0.35 | 0.68 | 0.67 | 0.53 | 0.90 | 0.70 | 0.62 | 0.61 | 0.24 | 0.53 | 0.98 (0.92-1.04, 0.54)^3^ |
|  | 9 (2.0) | 5 (1.1) | 10 (2.3) | 10 (2.0) | 8 (1.7) | 14 (2.5) | 11 (2.2) | 10 (2.2) | 10 (3.9) | 4 (1.8) | 9 (2.3) | 1.04 (0.98-1.12, 0.19)^4^ |
| NVT^6^ |  |  |  |  |  |  |  |  |  |  |  |  |
| 10B | 0.07 | 0.14 | 0.20 | 0.27 | 0.13 | 0.13 | 0.06 | 0.25 | 0.12 | 0.06 | 0.12 | 0.97 (0.85-1.10, 0.63)^3^ |
|  | 1 (0.2) | 2 (0.4) | 3 (0.7) | 4 (0.8) | 2 (0.4) | 2 (0.4) | 1 (0.2) | 4 (0.9) | 2 (0.8) | 1 (0.5) | 2 (0.5) | 1.03 (0.90-1.18, 0.65)^4^ |
| 15A | 0.29 | 0.56 | 0.89 | 1.14 | 0.92 | 1.35 | 0.89 | 1.12 | 0.12 | 0.42 | 0.83 | 0.99 (0.94-1.05, 0.80)^3^ |
|  | 4 (0.9) | 8 (1.7) | 13 (3.0) | 17 (3.3) | 14 (2.9) | 21 (3.8) | 14 (2.8) | 18 (3.9) | 2 (0.8) | 7 (3.2) | 14 (3.6) | 1.06 (1.00-1.12, 0.05)^4^ |
| 16/F^5^ | 0.21 | 0.07 | 0.14 | 0.27 | 0.66 | 0.84 | 0.51 | 0.19 | 0.37 | 0.18 | 0.24 | 1.02 (0.94-1.11, 0.66)^3^ |
|  | 3 (0.7) | 1 (0.2) | 2 (0.5) | 4 (0.8) | 10 (2.1) | 13 (2.3) | 8 (1.6) | 3 (0.7) | 6 (2.4) | 3 (1.4) | 4 (1.0) | 1.09 (1.00-1.19, 0.05)^4^ |
| 17F | 0.29 | 0.42 | 0.34 | 0.20 | 0.46 | 0.19 | 0.38 | 0.31 | 0.00 | 0.24 | 0.35 | 0.96 (0.88-1.05, 0.40)^3^ |
|  | 4 (0.9) | 6 (1.3) | 5 (1.1) | 3 (0.6) | 7 (1.4) | 3 (0.5) | 6 (1.2) | 5 (1.1) | 0 (0.0) | 4 (1.8) | 6 (1.6) | 1.02 (0.93-1.12, 0.64)^4^ |
| 20 | 0.07 | 0.21 | 0.14 | 0.80 | 0.33 | 0.77 | 0.32 | 0.19 | 0.06 | 0.18 | 0.00 | 0.93 (0.85-1.02, 0.11)^3^ |
|  | 1 (0.2) | 3 (0.7) | 2 (0.5) | 12 (2.4) | 5 (1.0) | 12 (2.2) | 5 (1.0) | 3 (0.7) | 1 (0.4) | 3 (1.4) | 0 (0.0) | 0.98 (0.89-1.08, 0.73)^4^ |
| 23A | 0.29 | 0.56 | 1.16 | 0.87 | 0.66 | 1.23 | 0.95 | 1.18 | 0.31 | 0.30 | 0.35 | 0.96 (0.91-1.02, 0.21)^3^ |
|  | 4 (0.9) | 8 (1.7) | 17 (3.9) | 13 (2.5) | 10 (2.1) | 19 (3.4) | 15 (3.0) | 19 (4.2) | 5 (2.0) | 5 (2.3) | 6 (1.6) | 1.03 (0.97-1.09, 0.40)^4^ |
| 23B | 0.50 | 0.35 | 0.61 | 0.94 | 0.59 | 0.90 | 0.82 | 0.50 | 0.74 | 0.84 | 1.06 | 1.06 (1.00-1.12, 0.05)^3^ |
|  | 7 (1.6) | 5 (1.1) | 9 (2.1) | 14 (2.7) | 9 (1.9) | 14 (2.5) | 13 (2.6) | 8 (1.8) | 12 (4.7) | 14 (6.4) | 18 (4.7) | 1.14 (1.07-1.21, <0.001)^4^ |
| 24/F^5^ | 0.36 | 0.63 | 0.68 | 1.20 | 0.72 | 0.58 | 0.38 | 0.50 | 0.25 | 0.12 | 0.30 | 0.90 (0.84-0.97, 0.003)^3^ |
|  | 5 (1.1) | 9 (2.0) | 10 (2.3) | 18 (3.5) | 11 (2.3) | 9 (1.6) | 6 (1.2) | 8 (1.8) | 4 (1.6) | 2 (0.9) | 5 (1.3) | 0.95 (0.89-1.02, 0.20)^4^ |
| 31 | 0.21 | 0.14 | 0.34 | 0.60 | 0.66 | 0.58 | 0.25 | 0.25 | 0.18 | 0.06 | 0.00 | 0.90 (0.82-0.99, 0.03)^3^ |
|  | 3 (0.7) | 2 (0.4) | 5 (1.1) | 9 (1.8) | 10 (2.1) | 9 (1.6) | 4 (0.8) | 4 (0.9) | 3 (1.2) | 1 (0.5) | 0 (0.00) | 0.95 (0.87-1.05, 0.33)^4^ |
| 35B | 0.14 | 0.00 | 0.14 | 0.33 | 0.20 | 0.45 | 0.32 | 0.25 | 0.31 | 0.12 | 0.18 | 1.04 (0.94-1.15, 0.44)^3^ |
|  | 2 (0.5) | 0 (0.0) | 2 (0.5) | 5 (1.0) | 3 (0.6) | 7 (1.3) | 5 (1.0) | 4 (0.9) | 5 (2.0) | 2 (0.9) | 3 (0.8) | 1.12 (1.00-1.24, 0.04)^4^ |
| 35F | 0.36 | 0.56 | 0.27 | 0.87 | 0.66 | 0.65 | 0.51 | 0.69 | 0.49 | 0.30 | 0.77 | 1.02 (0.95-1.09, 0.59)^3^ |
|  | 5 (1.1) | 8 (1.7) | 4 (0.9) | 13 (2.5) | 10 (2.1) | 10 (1.8) | 8 (1.6) | 11 (2.4) | 8 (3.1) | 5 (2.3) | 13 (3.4) | 1.09 (1.02-1.16, 0.01)^4^ |
| 38 | 0.36 | 0.21 | 0.27 | 0.33 | 0.59 | 0.26 | 0.19 | 0.31 | 0.25 | 0.00 | 0.06 | 0.90 (0.81-0.99, 0.03)^3^ |
|  | 5 (1.1) | 3 (0.7) | 4 (0.9) | 5 (1.0) | 9 (1.9) | 4 (0.7) | 3 (0.6) | 5 (1.1) | 4 (1.6) | 0 (0.0) | 1 (0.3) | 0.95 (0.85-1.05, 0.29)^4^ |
| 6C | 0.64 | 1.81 | 0.55 | 1.14 | 0.92 | 0.90 | 0.63 | 0.62 | 0.43 | 0.36 | 0.24 | 0.89 (0.84-0.94, <0.001)^3^ |
|  | 9 (2.0) | 26 (5.7) | 8 (1.8) | 17 (3.3) | 14 (2.9) | 14 (2.5) | 10 (2.0) | 10 (2.2) | 7 (2.7) | 6 (2.8) | 4 (1.0) | 0.93 (0.88-0.99, 0.02)^4^ |
| 7 | 0.14 | 0.21 | 0.00 | 0.07 | 0.07 | 0.06 | 0.32 | 0.25 | 0.18 | 0.06 | 0.47 | 1.14 (1.01-1.29, 0.04)^3^ |
|  | 2 (0.5) | 3 (0.7) | 0 (0.0) | 1 (0.2) | 1 (0.2) | 1 (0.2) | 5 (1.0) | 4 (0.9) | 3 (1.2) | 1 (0.5) | 8 (2.1) | 1.23 (1.08-1.40, 0.001)^4^ |
| 9N | 1.00 | 1.12 | 1.09 | 1.40 | 1.97 | 2.71 | 2.16 | 2.30 | 1.23 | 1.14 | 1.36 | 1.02 (0.98-1.06, 0.29)^3^ |
|  | 14 (3.2) | 16 (3.5) | 16 (3.6) | 21 (4.1) | 30 (6.2) | 42 (7.5) | 34 (6.9) | 37 (8.1) | 20 (7.8) | 19 (8.7) | 23 (6.0) | 1.09 (1.05-1.14, <0.001)^4^ |
| Other^7^ | 0.64 | 0.56 | 1.23 | 0.60 | 0.46 | 0.32 | 0.57 | 0.19 | 0.37 | 0.18 | 0.59 | 0.91 (0.85-0.98, 0.009)^3^ |
|  | 9 (2.0) | 8 (1.7) | 18 (4.1) | 9 (1.8) | 7 (1.4) | 5 (0.9) | 9 (1.8) | 3 (0.7) | 6 (2.4) | 3 (1.4) | 10 (2.6) | 0.97 (0.90-1.04, 0.35)^4^ |
| ^1^Poisson Regression | | | | | | | | | | | | |
| ^2^Pneumococcal conjugate vaccine | | | | | | | | | | | | |
| ^3^N = 65+ age-group population | | | | | | | | | | | | |
| ^4^N = total number of cases from 65+ age-group with serotype known | | | | | | | | | | | | |
| ^5^15B/C = 15B, 15C. 16/F = 16, 16F. 24/F = 24, 24F. | | | | | | | | | | | | |
| ^6^Non-vaccine serotypes, i.e. not covered by PCV20 | | | | | | | | | | | | |
| ^7^Non-vaccine serotypes with overall proportion < 0.5% combined to other: non-typable, 10, 11, 12, 13, 15, 15F, 18, 18F, 19, 2, 21, 22, 25, 27, 28, 29, 33, 33A, 34, 35, 36, 37, 39, 41, 42, 6, 6D, 9 | | | | | | | | | | | | |

**Supplementary figure**: Introduction of pneumococcal conjugate vaccines


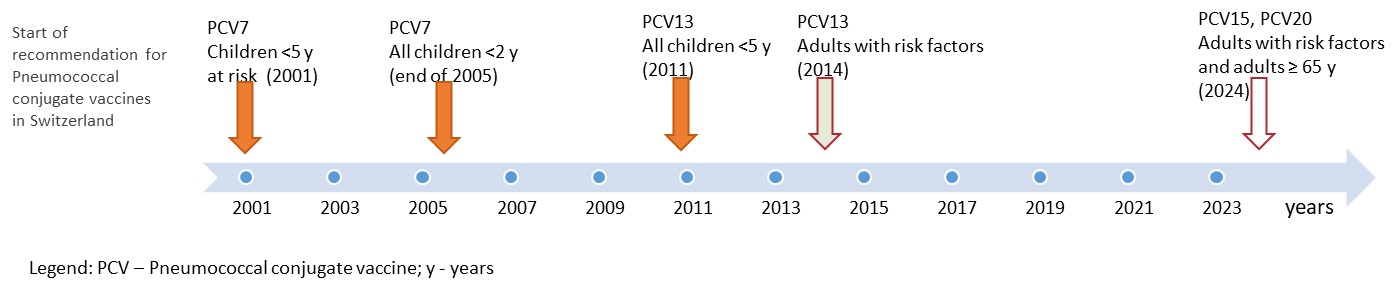


Risk factors for adult vaccination include:

- Heart failure; Chronic obstructive pulmonary disease; Severe asthma (patients with prolonged or frequent treatment with oral corticosteroids); Bronchiectasis due to antibody deficiency; Liver cirrhosis; Asplenia (anatomic or functional); Renal failure (clearance 30mg/min); Nephrotic syndrome; Sickle cell anemia; Diabetes mellitus, poorly controlled with heart or renal failure; Lymphoma, leukemia, myeloma; Solid organ transplantation (candidates and recipients); Stem cell transplantation recipients; Autoimmune diseases, which likely require immunosuppression; Iatrogenic immunosuppression (including systemic longterm treatment with corticosteroids or radiation therapy); HIV infection; Congenital immunodeficiency, common variable immunodeficiency syndrome, polysaccharide antibody deficiency; Mannose-binding lectin deficiency; Preterm delivery (birth prior to 33^rd^ week or birth weight < 1500g); Cochlea implant (present or planned); Basal skull fracture or deformity , cerebrospinal fistula
